# The individual and combined impacts of pre-existing diabetes and dementia on ischemic stroke outcomes: a registry-based cohort study

**DOI:** 10.1186/s12872-024-04050-3

**Published:** 2024-07-30

**Authors:** Kyi Lae Shune Kyaw, Tiberiu A. Pana, Joao H. Bettencourt-Silva, Anthony K. Metcalf, Phyo K. Myint, John F. Potter

**Affiliations:** 1 Edinburgh Medical School, University of Edinburgh, Edinburgh, Scotland, UK; 2 Aberdeen Cardiovascular & Diabetes Centre, School of Medicine, Medical Sciences & Nutrition, University of Aberdeen, Aberdeen, Scotland, UK; 3 Ageing Clinical and Experimental Research Team, Institute of Applied Health Sciences, University of Aberdeen, Aberdeen, Scotland, UK; 4 Norfolk & Norwich University Hospitals NHS Foundation Trust, Norwich, UK; 5 Healthcare and Life Sciences, IBM Research, Norwich, UK; 6 Aberdeen Royal Infirmary, NHS Grampian, Aberdeen, Scotland, UK; 7 Norwich Medical School, University of East Anglia, Norwich, UK

**Keywords:** Acute ischaemic stroke, Diabetes, Dementia, Outcomes

## Abstract

**Background:**

Individually, diabetes mellitus and dementia are associated with poorer outcomes after stroke. However, the combined impact of these pre-existing factors on acute ischemic stroke (AIS) outcomes has not been examined.

**Methods:**

All consecutive patients with AIS admitted to Norfolk and Norwich University Hospitals between 2003 and 2016 (catchment population ~ 900,000) were divided into four groups: those with neither diabetes nor dementia (reference), with diabetes without dementia, with dementia without diabetes, and with both co-morbidities. In-hospital mortality, length of hospital stay (LoS), and disability outcomes were analysed using logistic regressions. Post-discharge mortality and recurrence were assessed using Cox regressions. Additionally, interaction terms were added to the models for the short-term outcomes and long-term mortality to test for synergistic effects of diabetes and dementia. Models were adjusted for age, sex, Oxfordshire Community Stroke Project classification, comorbidities, hematological and biochemical measures, and antithrombotic medications.

**Results:**

The cohort was 10,812 patients with 52% females and a median age of 80. The median follow-up was 3.8 years for stroke recurrence and 5.5 years for mortality. No significant differences between the four groups existed for in-hospital mortality and post-stroke disability. Patients with dementia had significantly longer LoS (OR 2.25 [95% CI: 1.34–3.77] and 1.31 [1.02–1.68] with and without diabetes, respectively). Patients with both comorbidities had the highest risk of stroke recurrence (HR 2.06 [1.12–3.77]), followed by those with only dementia (1.59 [1.15–2.20]) and only diabetes (1.25 [1.06–1.49]). Similarly, the patient group with both diabetes and dementia had the highest long-term mortality risk (1.76 [1.33–2.37]). The hazard ratios for patients with only dementia and only diabetes were 1.71 [1.46–2.01] and 1.19 [1.08–1.32], respectively. No significant interactions were seen between diabetes and dementia with regards to their effects on the outcomes.

**Conclusion:**

Individual and cumulative impacts of the two conditions on long-term mortality and stroke recurrence were notable. However, no synergistic impact of the two comorbidities were seen on the stroke outcomes tested in our study. Therefore, tailoring the management of stroke patients based on additional requirements associated with each pre-existing condition will be more impactful towards improving outcomes.

**Supplementary Information:**

The online version contains supplementary material available at 10.1186/s12872-024-04050-3.

## Background

Diabetes mellitus (referred to as diabetes in this paper) and dementia are two groups of conditions with high disease burdens and are both linked to poorer ischemic stroke outcomes. Previous research has shown pre-existing diabetes to be associated with increased stroke mortality [1], recurrence, disability, and in-hospital mortality [2–4]. Similarly, pre-existing dementia has been shown to increase the risks of mortality and poor functional outcomes in stroke patients [5, 6].

Despite the frequent coexistence of these conditions [7], the combined impact of pre-existing diabetes and dementia on stroke outcomes has not been studied. Previous research on the interactions between the three conditions has mainly focused on associations between baseline diabetes and increased risk of post-stroke dementia [8]. Various processes have been suggested as common mechanisms between diabetes and different types of dementia [9–11]. These include cerebrovascular damage resulting from atherosclerotic build up in vessels secondary to diabetes and in vascular dementia, as well as mechanisms associated with the role of glycation end products from oxidative stress, which has been found to play a role in diabetes, dementia, and ischaemic strokes. Furthermore, studies have proposed that diabetes affects cognitive function through both Aβ/tau-dependent and independent mechanisms [12]. There is also evidence showing differences in post-stroke care, such as reduced administration of thrombolysis and prescription of cardiovascular disease (CVD) prevention medications to patients with diabetes or dementia. Consequently, this suggests reason for a possible cumulative impact of having both conditions on stroke outcomes [13, 14].

Against this background, we aimed to investigate the individual as well as the combined impacts of dementia and diabetes on short-term (in-hospital death, length of stay at hospital (LoS), and excess disability) and long-term (mortality and ischemic stroke recurrence) outcomes using a cohort of patients with acute ischemic stroke (AIS) extracted from a UK-based registry.

## Methods

### Study design and participants

The study population consisted of patients with acute ischemic stroke and was drawn from the Norfolk and Norwich Stroke and TIA register (NNSTR) – a prospectively collected UK regional stroke register in the East Anglia region. The register includes all stroke admissions to the Norfolk and Norwich University Hospital, which is a tertiary referral center in England with a catchment population of approximately 900,000 (2017) [15]. The details of data collection methods have been previously reported [16]. The register received ethical approval from the Newcastle and Tyneside National Health Service (NHS) and Research Ethics Committee (17/NE/0277) as a research database and therefore did not require individual patient consent. The study protocol was approved by the Steering Committee of the Register.

Patients admitted with AIS between January 2003 and December 2016 were included in the study population. For all participants, AIS was diagnosed based on patient history, examination, and computerized tomography/magnetic resonance imaging results. Follow-up data were collected in June 2017 and the maximum follow-up was 14.4 years. Due to record linkage with the NHS system in the UK, the database has ascertainment of comorbidities and almost complete follow-up with only 0.90% (78 of 8692 patients) of the sample being lost to follow-up for the post-discharge mortality and recurrence analysis.

**Fig. 1:**
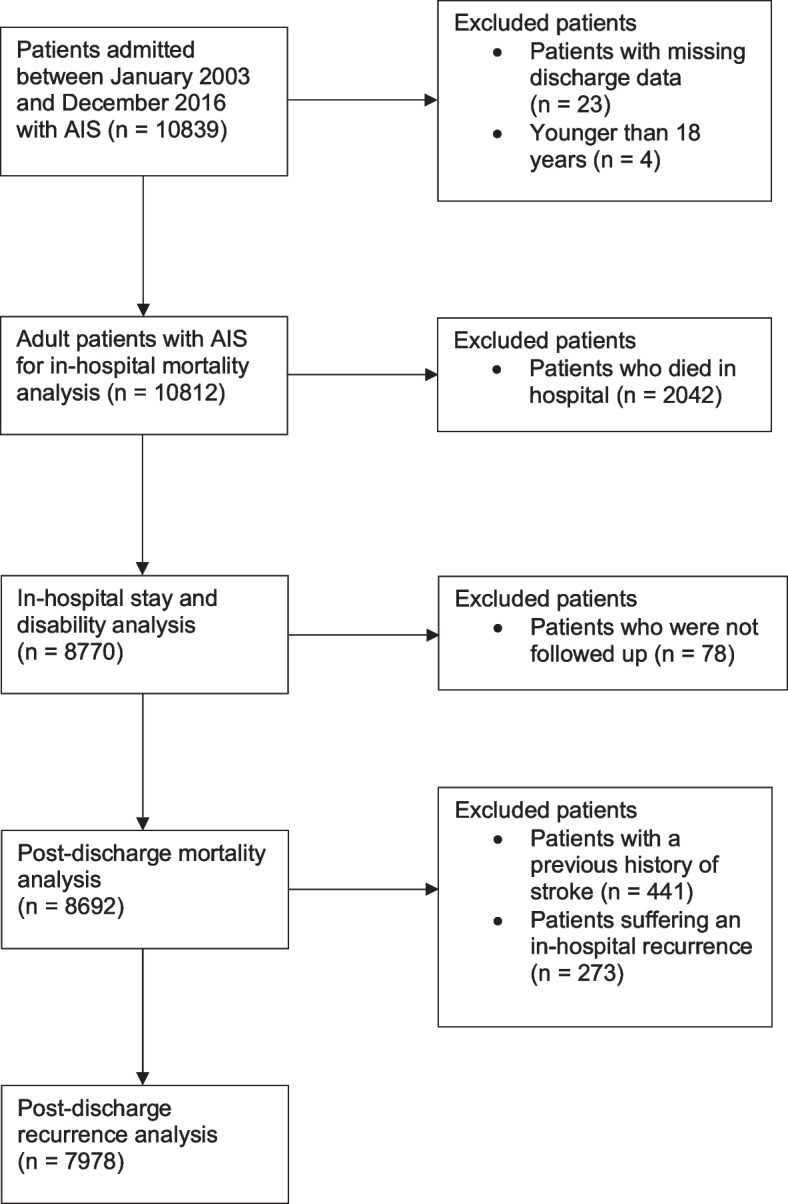
Patient population flowchart showing selection criteria for each stage of analysis

The exclusion criteria, outcomes of interests (in-hospital mortality, LoS, excess disability, post-discharge mortality, and stroke recurrence), and covariates were all agreed upon a priori. The exclusion criteria were applied sequentially for the various stages of the analysis, according to the outcomes assessed at each stage (Fig. 1). An initial population of 10,839 patients were extracted from the database. After excluding patients with missing discharge data (*n* = 23) and patients younger than 18 (*n* = 4), a population of 10,812 adult patients were eligible to be included.

### Exposure groups

The exposure groups (pre-existing dementia and diabetes), diagnosed by the clinical teams at the tertiary center, were identified from the NNSTR using the *10th Revision of the International Classification of Disease (ICD-10)* codes. Dementia included Alzheimer’s disease (F00), vascular dementia (F01), dementia in other diseases classified elsewhere (F02), unspecified dementia (F03), and delirium due to psychological condition (F05). Delirium due to psychological condition (F05) is included as part of the dementia subgroups due to coding system used in the registry, which grouped F05 with F02 and F03 under ‘other types of dementia’. As for diabetes, Type 1 (E10), Type 2 (E11), malnutrition-related (E12), other specified (E13), and unspecified diabetes mellitus (E14) were included. Our database, which had electronic record linkage with primary care comorbidity data, extracted any diagnoses of dementia and diabetes before, during, and after stroke admission. Our study defined pre-existing dementia and diabetes as conditions diagnosed before stroke admission or during the hospital stay. Patients were then split into four mutually exclusive categories: neither pre-existing dementia nor diabetes, pre-existing diabetes but not dementia, pre-existing dementia but not diabetes, and both pre-existing dementia and diabetes.

### Confounder selection

Potential confounders were selected based on existing literature [1, 17–20]. Our analyses included age, sex, Oxfordshire Community Stroke Project classification (OCSP), pre-stroke modified Rankin scale (mRS), comorbidities, antithrombotic medications, and relevant biochemical and hematological measurements on admission (random plasma glucose, creatinine, sodium, hemoglobin, white cell count, and platelets) as the variables. Biochemical and hematological measurements were collected by electronic record linkage. Comorbidities that were included in the analyses (pneumonia, asthma, COPD, history of transient ischemic attack [TIA], hemorrhagic or other types of stroke, myocardial infarction, hyperlipidemia, peripheral vascular disease, heart failure, atrial fibrillation, hypertension, cancers, chronic kidney disease, and liver disease) were identified using ICD-10 codes. The codes can be found in Additional file 1. Any diagnosis of the comorbidities occurring before, during, and after AIS admission were extracted from our database.

### Study outcomes

Data for the five outcomes of interest were extracted from the NNSTR. Following exclusion of patients with missing discharge data and patients < 18 years, a total of 10,812 patients were included in the in-hospital mortality analysis. 2,042 patients who died in hospital were then excluded and the remaining 8,770 patients were included in the analyses of the other short-term outcomes (LoS and excess disability). Binomial logistic regressions were performed to assess in-hospital mortality and LoS longer than the median among the four exposure groups. Both models included age, sex, OCSP, comorbidities, admission antithrombotic medications, and admission biochemical measures as covariates. A multinomial logistic regression was used to compare excess disability between the four groups. The difference between the mRS measures ($\Delta$ mRS) at admission and at discharge was calculated. The resulting values were then split into tertiles, which were used as the outcome for the multinomial logistic regression. Alongside the specified confounders in the previous two models, the pre-stroke mRS was also included in the model assessing excess disability.

After excluding patients who were not followed up, the recorded dates of death from the database were used to analyse 8,692 patients for post-discharge mortality. 714 more patients were excluded for the post-discharge stroke recurrence outcome due to previous history of stroke and stroke recurrence during hospital stay. This led to 7,978 patients being analysed for long-term recurrence, using information on readmission with AIS. The two post-discharge outcomes were assessed using Cox regression models. Cause specific hazard ratios were determined for the recurrence outcome given the competing risk of death. The full exclusion criteria applied at each stage is detailed in Fig. 1.

Where possible, for each model of the study outcomes, an additional interaction test was carried out. Pre-existing diabetes and pre-existing dementia were included as interaction terms to explore any interactive effects on the short-term and the long-term mortality outcomes. An interaction analysis was not carried out for the post-discharge recurrence outcome due to small numbers in the subgroups.

### Statistical analysis

The software Stata 14.1 (StataCorp 2015, Statistical Software; Release 14, College Station, TX: StataCorp LP) was used to perform statistical analysis. Pearson’s Chi-Square test was used to compare the categorical variables between the exposure groups. One-way ANOVA and Kruskal–Wallis tests were used to compare normally distributed and non-normally distributed numerical variables, respectively.

### Missing data

The following key variables contained missing data: OCSP, pre-stroke mRS, post-stroke mRS, random plasma glucose, creatinine, sodium, hemoglobin, white cell count, and platelets at admission. Frequencies of missing data can be found in Additional file 2. After comparing patients with missing data to those without missing data (see tables in Additional file 3), we found that patients with missing data were more likely to have a shorter LoS and a lower comorbidity burden. This suggests that the data were missing-at-random [21]. We performed multiple imputation by chained equations with 20 imputations using predictive mean matching for all the variables. Due to the high percentage of the missing National Institutes of Health Stroke Scale (NIHSS) variable, sensitivity analyses using multiply imputed NIHSS values were performed as separate models for each outcome. The analyses did not show any significant differences between the two models. Therefore, the results presented in the paper are from NIHSS-adjusted models with the exception of interactions tests, which are presented from non-adjusted models.

## Results

### Descriptive statistics

Table 1 displays the characteristics of the patients, stratified by their pre-existing condition status. For variables with missing values, statistics for the complete cases are presented and the corresponding missing data information for each is provided. The median (interquartile range [IQR]) age of the cohort was 80.00 years (71.63–86.00) and 48% were males. The median follow-up was 2,001 days (5.5 years) for mortality and 1,405 days (3.8 years) for recurrence. Out of 10,812 patients, 8,579 (79.3%) had neither comorbidity, 1,873 (17.3%) had diabetes only, 287 (2.7%) had dementia only, and 73 (0.7%) had both diabetes and dementia. Patients with isolated dementia were the oldest age group with a median of 86.00 years (IQR 81.00–90.00) while patients with isolated diabetes were the youngest with a median of 79.00 years (IQR 71.00–85.00). The highest comorbidity burden was observed in patients with both dementia and diabetes, followed by patients with only diabetes, only dementia, and neither comorbidity. The highest in-hospital mortality rate was observed in patients with only dementia and with both pre-existing conditions at 29.3%. This was followed by patients with neither comorbidity at 18.7%, and patients with diabetes only at 17.7%.

**Table 1:** Characteristics of ischemic stroke patients, stratified by pre-existing condition

	**Total**	**Neither DM or dementia**	**DM only**	**Dementia only**	**Both DM and dementia**	***p*-value**
N	10,812	8579	1873	287	73	
Age, median(IQR)	80.00 (71.63–86.00)	80.00 (71.00–86.41)	79.00 (71.00–85.00)	86.00 (81.00–90.00)	84.00 (79.00–87.53)	< 0.001
Sex, n (%)						< 0.001
Male	5189 (47.99)	4008 (46.72)	1027 (54.83)	113 (39.37)	41 (39.37)	
Female	5623 (52.01)	4571 (53.28)	846 (45.17)	174 (60.63)	32 (60.63)	
**OCSP classification, n (%)**						**0.001**
LACS	2484 (22.97)	1965 (22.90)	446 (23.81)	60 (20.91)	13 (20.91)	
PACS	3680 (34.04)	2904 (33.85)	644 (34.38)	104 (36.24)	28 (36.24)	
POCS	1611 (14.90)	1277 (14.89)	299 (15.96)	28 (9.76)	7 (9.76)	
TACS	1954 (18.07)	1580 (18.42)	306 (16.34)	54 (18.82)	14 (18.82)	
Unknown	379 (3.51)	306 (3.57)	48 (2.56)	19 (6.62)	6 (6.62)	
*Missing, n (%)*	704 (6.51)	547 (6.38)	130 (6.94)	22 (7.67)	5 (6.85)	0.690
**In-hospital bloods at admission**						
Random plasma glucose, median (IQR)	6.30 (5.50–7.80)	6.10 (5.40–7.30)	8.50 (6.30–11.90)	6.10 (5.30–7.40)	7.30 (6.20–11.30)	< 0.001
*Missing, n (%)*	2603 (24.08)	2132 (24.85)	383 (20.45)	73 (25.44)	15 (20.55)	< 0.001
Fasting glucose, median (IQR)	5.50 (5.00–6.40)	5.40 (4.90–6.10)	7.70 (6.00–10.00)	5.50 (4.80–6.30)	6.80 (4.85–12.75)	< 0.001
*Missing, n (%)*	9424 (87.16)	7395 (86.20)	1690 (90.23)	270 (94.08)	69 (94.52)	< 0.001
Creatinine, median (IQR)	86.00 (71.00–108.00)	85.00 (71.00–106.00)	90.00 (73.00–118.00)	87.50 (71.50–111.50)	93.00 (72.00–117.00)	< 0.001
*Missing, n (%)*	297 (2.75)	239 (2.79)	49 (2.62)	7 (2.44)	2 (2.74)	0.965
Sodium, mean (SD)	138.30 (4.23)	138.40 (4.14)	137.73 (4.27)	138.82 (6.00)	139.29 (5.17)	< 0.001
*Missing, n (%)*	309 (2.86)	251 (2.93)	48 (2.56)	7 (2.44)	3 (4.11)	0.724
Albumin, mean (SD)	36.63 (5.43)	36.79 (5.39)	36.21 (5.56)	34.97 (5.01)	34.79 (5.47)	< 0.001
*Missing, n (%)*	467 (4.32)	384 (4.48)	70 (3.74)	10 (3.48)	3 (4.11)	0.469
Cholesterol, mean (SD)	4.85 (1.31)	4.92 (1.30)	4.55 (1.33)	4.85 (1.24)	4.62 (1.32)	< 0.001
*Missing, n (%)*	3846 (35.57)	3004 (35.02)	680 (36.31)	130 (45.30)	32 (43.84)	0.001
INR, median (IQR)	1.04 (0.98–1.13)	1.04 (0.98–1.12)	1.04 (0.98–1.14)	1.05 (0.99–1.14)	1.06 (1.00–1.13)	0.094
*Missing, n (%)*	1554 (14.37)	1257 (14.65)	240 (12.81)	49 (17.07)	8 (10.96)	0.084
CRP, median (IQR)	11.00 (5.00–36.00)	12.00 (5.00–37.00)	10.00 (4.00–31.00)	13.00 (4.00–38.00)	14.50 (6.50–42.00)	0.042
*Missing, n (%)*	1872 (17.31)	1581 (18.43)	255 (13.61)	31 (10.80)	5 (6.85)	< 0.001
Hemoglobin, mean (SD)	134.61 (19.57)	135.35 (19.47)	131.68 (19.75)	132.67 (19.58)	130.00 (19.03)	< 0.001
*Missing, n (%)*	556 (5.14)	432 (5.04)	107 (5.71)	12 (4.18)	5 (6.85)	0.488
White cell count, median (IQR)	8.80 (7.10–11.30)	8.80 (7.10–11.20)	9.00 (7.40–11.70)	8.90 (7.10–11.00)	9.00 (7.60–11.60)	< 0.001
*Missing, n (%)*	268 (2.48)	215 (2.51)	44 (2.35)	7 (2.44)	2 (2.74)	0.981
Platelet count, median (IQR)	245.00 (201.00–302.00)	245.00 (201.00–301.00)	245.00 (199.00–305.00)	248.00 (201.50–310.00)	263.00 (209.00–321.00)	0.431
*Missing, n (%)*	283 (2.62)	228 (2.66)	46 (2.46)	7 (2.44)	2 (2.74)	0.963
**Antithrombotic medications, n (%)**						
Antiplatelets at admission	3610 (33.39)	2754 (32.10)	719 (38.39)	113 (39.37)	24 (39.37)	< 0.001
Antiplatelets at discharge	7045 (65.16)	5629 (65.61)	1223 (65.30)	154 (53.66)	39 (53.66)	< 0.001
Anticoagulants at admission	169 (1.56)	120 (1.40)	41 (2.19)	3 (1.05)	5 (1.05)	< 0.001
Anticoagulants at discharge	434 (4.01)	322 (3.75)	87 (4.64)	20 (6.97)	5 (6.97)	0.009
**Pre-existing comorbidities**						
Pneumonia, n (%)	2000 (18.50)	1477 (17.22)	410 (21.89)	83 (28.92)	30 (28.92)	< 0.001
Asthma, n (%)	1011 (9.35)	753 (8.78)	225 (12.01)	25 (8.71)	8 (8.71)	< 0.001
Chronic obstructive pulmonary disease, n (%)	897 (8.30)	680 (7.93)	183 (9.77)	28 (9.76)	6 (9.76)	0.053
Transient ischemic attack, n (%)	559 (5.17)	415 (4.84)	112 (5.98)	26 (9.06)	6 (9.06)	0.002
Myocardial Infarction, n (%)	811 (7.50)	588 (6.85)	188 (10.04)	27 (9.41)	8 (9.41)	< 0.001
Hyperlipidemia, n (%)	1469 (13.59)	1050 (12.24)	383 (20.45)	26 (9.06)	10 (9.06)	< 0.001
Congenital heart disease, n (%)	3004 (27.78)	2136 (24.90)	737 (39.35)	98 (34.15)	33 (34.15)	< 0.001
Peripheral vascular disease, n (%)	452 (4.18)	276 (3.22)	159 (8.49)	11 (3.83)	6 (3.83)	< 0.001
Heart failure, n (%)	1543 (14.27)	1117 (13.02)	362 (19.33)	49 (17.07)	15 (17.07)	< 0.001
Atrial fibrillation, n (%)	3537 (32.71)	2752 (32.08)	625 (33.37)	126 (43.90)	34 (43.90)	< 0.001
Hypertension, n (%)	6592 (60.97)	4943 (57.62)	1424 (76.03)	172 (59.93)	53 (59.93)	< 0.001
Cancers, n (%)	1771 (16.38)	1407 (16.40)	313 (16.71)	39 (13.59)	12 (13.59)	0.618
Chronic kidney disease, n (%)	719 (6.65)	448 (5.22)	231 (12.33)	21 (7.32)	19 (7.32)	< 0.001
Liver disease, n (%)	170 (1.57)	101 (1.18)	65 (3.47)	2 (0.70)	2 (0.70)	< 0.001
Charlson comorbidity index, median (IQR)	3.00 (1.00–4.00)	2.00 (1.00–4.00)	4.00 (2.00–6.00)	3.00 (2.00–4.00)	5.00 (3.00–7.00)	< 0.001
**Outcomes**						
In-hospital mortality, n (%)	2042 (18.89)	1605 (18.71)	332 (17.73)	84 (29.27)	21 (29.27)	< 0.001
Length of stay, median (IQR)	8.00 (3.00–17.00)	7.66 (3.00–16.28)	8.00 (3.16–18.00)	12.00 (5.00–25.00)	16.00 (7.00–33.00)	< 0.001
mRS admission, median (IQR)	0.00 (0.00–2.00)	0.00 (0.00–1.00)	0.00 (0.00–2.00)	3.00 (1.00–4.00)	2.00 (1.00–3.00)	< 0.001
*Missing, n (%)*	607 (5.61)	485 (5.65)	99 (5.29)	19 (6.62)	4 (5.48)	0.812
mRS discharge, median (IQR)	3.00 (1.00–6.00)	3.00 (1.00–6.00)	3.00 (1.00–5.00)	4.00 (3.00–6.00)	4.00 (3.00–6.00)	< 0.001
*Missing, n (%)*	3157 (29.20)	2650 (30.89)	453 (24.19)	44 (15.33)	10 (13.70)	< 0.001
$\Delta$ mRS, median (IQR)	2.00 (0.00–3.00)	2.00 (0.00–4.00)	2.00 (0.00–3.00)	1.00 (0.00–3.00)	1.00 (0.00–3.00)	0.006
*Missing, n (%)*	3557 (32.90)	2975 (34.68)	514 (27.44)	56 (19.51)	12 (16.44)	< 0.001
Total NIHSS	4.00 (1.00–10.00)	4.00 (1.00–9.00)	4.00 (1.00–11.00)	4.00 (1.00–12.00)	8.00 (2.00–13.00)	0.317
*Missing, n (%)*	9290 (85.92)	7444 (86.77)	1572 (83.93)	219 (76.31)	55 (75.34)	< 0.001

The one-way ANOVA, Kruskal–Wallis, and Pearson’s chi-square test were used to find the differences between groups for the normally distributed, non-normally distributed, and categorical variables respectively

*DM* indicates diabetes mellitus, *OCSP* Oxfordshire Community Stroke Project, *LACS* lacunar stroke, *PACS* partial anterior circulation stroke, *POCS* posterior circulation stroke, *TACS* total anterior circulation stroke, *NIHSS* National Institutes of Health Stroke Scale, *mRS* modified Rankin scale, *IQR* interquartile range, *INR* international normalized ratio, *CRP* C-reactive protein

### Short-term outcomes

The results of the in-hospital mortality, LoS, and excess disability analyses are presented in Table 2. Results yielded from further analyses that test for interaction are presented in Additional file 6. When compared to the patient group without either comorbidity (reference group), there was no significant increase in odds of in-hospital death for patients in any of the comorbidity groups.

**Table 2:** Results of in-hospital outcomes analyses

	OR [95% CI]	*P* value
**In-hospital death**		
No DM or dementia	1 (reference)	
DM only	0.84 [0.71–1.00]	0.053
Dementia only	1.23 [0.90–1.68]	0.196
Both DM and dementia	1.13 [0.59–2.17]	0.711
**LoS greater than median**		
No DM or dementia	1 (reference)	
DM only	1.12 [1.00–1.26]	0.052
Dementia only	1.31 [1.02–1.68]	0.031
Both DM and dementia	2.25 [1.34–3.77]	0.002
**Excess disability**		
**Second** $\boldsymbol{\Delta }$ **mRS tertile vs first** $\boldsymbol{\Delta }$ **mRS tertile**		
No DM or dementia	1 (reference)	
DM only	0.99 [0.85–1.16]	0.907
Dementia only	1.26 [0.91–1.75]	0.169
Both DM and dementia	1.24 [0.65–2.39]	0.509
**Third** $\boldsymbol{\Delta }$ **mRS tertile vs first** $\boldsymbol{\Delta }$ **mRS tertile**		
No DM or dementia	1 (reference)	
DM only	1.00 [0.84–1.19]	0.971
Dementia only	1.54 [1.05–2.25]	0.027
Both DM and dementia	1.61 [0.76–3.42]	0.213

Table 2 shows the results of the logistic regression models with multiple imputed NIHSS adjustment; full table with and without NIHSS adjustments can be seen in Additional file 4; Models were also adjusted for age, sex, OCSP classification, mRS, comorbidities (pneumonia, asthma, COPD, transient ischemic attack [TIA], myocardial infarction, hyperlipidemia, peripheral vascular disease, heart failure, atrial fibrillation, hypertension, cancers, chronic kidney disease, liver disease, and hemorrhagic stroke, and other types of stroke), antithrombotic medications, and relevant biochemical and hematological measurements on admission (random plasma glucose, creatinine, sodium, hemoglobin, white cell count, and platelets)

*DM* indicates diabetes mellitus, *mRS* modified Rankin scale, *LoS* length of stay, *OR* odds ratio, *CI* confidence interval

The median for LoS was 8 days. In comparison to the reference group, patients with both dementia and diabetes had a 125% increase in odds of having an LoS greater than the median (odds ratio [95% CI] = 2.25 [1.34–3.77]) and patients with only dementia had a 31% increase. (1.31 [1.02–1.68]). Those with only diabetes did not have a significant increase in odds of having a LoS longer than the median (1.12 [1.00–1.26]). There was no significant synergistic impact of diabetes and dementia on LoS (1.52 [0.86–2.71]).

As for stroke-associated excess disability, membership of the first $\Delta$ mRS tertile served as the baseline. Having dementia but not diabetes was associated with an increased likelihood being in the third $\Delta$ mRS tertile (1.54 [1.05–2.25]). None of the comorbidity groups predicted membership of the second $\Delta$ mRS tertile. Interaction tests showed no significant synergistic increases in the likelihood of second or third $\Delta$ mRS tertile membership associated with the two comorbidities. For all three in-hospital outcomes, sensitivity analyses using multiply imputed NIHSS variables showed no significant differences (see Additional file 4 for results without NIHSS adjustment).

### Long-term outcomes

**Fig. 2:**
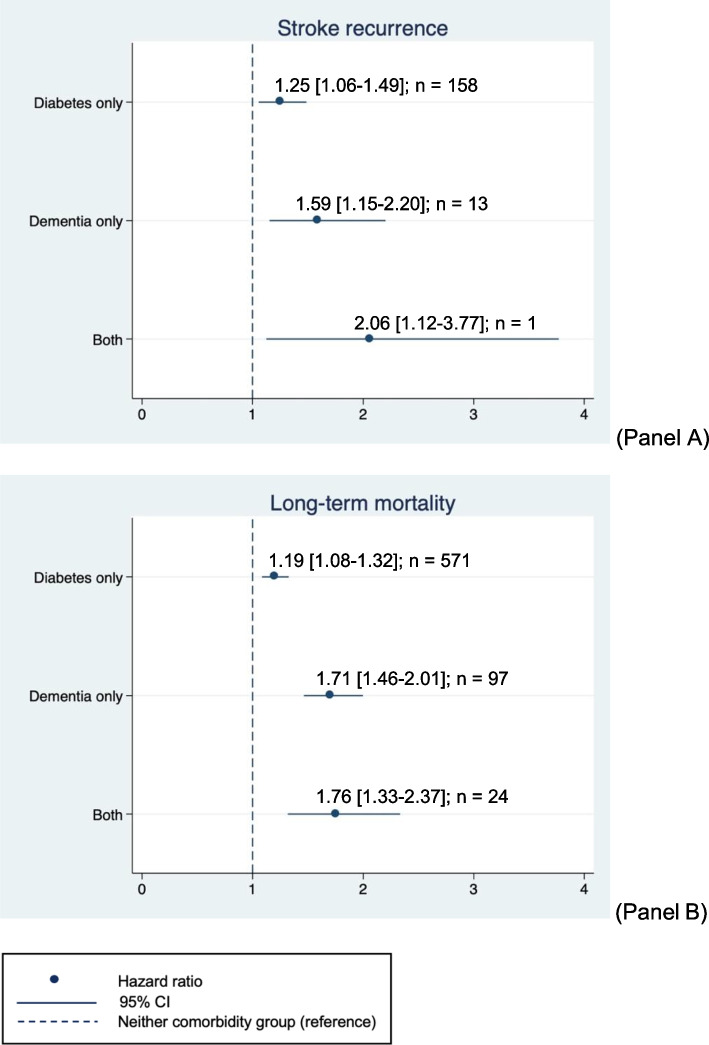
Graphical representations of long-term outcomes results. Legend: **A** Stroke recurrence (**B**) long-term mortality; The results demonstrated are yielded from Cox regression models with NIHSS adjustments. Models were also adjusted for age, sex, OCSP classification, mRS, comorbidities (pneumonia, asthma, COPD, transient ischemic attack [TIA], myocardial infarction, hyperlipidemia, peripheral vascular disease, heart failure, atrial fibrillation, hypertension, cancers, chronic kidney disease, liver disease, and hemorrhagic stroke, and other types of stroke), antithrombotic medications, and relevant biochemical and hematological measurements at discharge (random plasma glucose, creatinine, sodium, hemoglobin, white cell count, and platelets)

The results of the post-discharge long-term mortality and long-term ischemic stroke recurrence are detailed in Fig. 2 and Additional file 5. Post-discharge deaths were recorded and showed a total of 2,365 (34.2%) for patients with neither dementia or diabetes, 571 (37.2%) for those with diabetes only, 97 (47.8%) for those with dementia only, and 24 (49.0%) for those with both comorbidities. When compared to the reference group, the long-term mortality hazard ratio for the patient group with both comorbidities was highest (1.76 [1.33–2.37]). This was followed by those with isolated dementia (1.71 [1.46–2.01]) and with isolated diabetes (1.19 [1.08–1.32]).

Pertaining to long-term stroke recurrence that occurred during the follow-up period, 617 (9.7%), 158 (11.5%), 13 (6.9%), and 1 (2.6%) recurrence were recorded for patients with neither comorbidity, only diabetes, only dementia, and both diabetes and dementia, respectively. When compared to the reference group, the highest risk of recurrence was associated with the group with both comorbidities (2.06 [1.12–3.77]). Patients with only dementia but not diabetes had a 59% increased risk of recurrence (1.59 [1.15–2.20]), and those with only diabetes but not dementia had a 25% increased risk of recurrence (1.25 [1.06–1.49]). An interaction analysis of the Cox regression model for the long-term mortality showed no synergistic effects of diabetes and dementia (0.85 [0.61–1.18]). The complete table can be found in Additional file 7. Sensitivity analyses adjusting for multiply imputed NIHSS scores performed for both outcomes did not show any differences (see Additional file 5).

## Discussion

Using a large, prospectively collected real-world stroke registry, our study found increases in the stroke recurrence risk for patients with pre-existing diabetes and dementia. Increases in risk were seen in those with isolated diabetes, isolated dementia, and both conditions. While there are individual and combined impacts on recurrence risk, a synergistic impact could not be tested for this outcome due to sample sizes of some subgroups being small (*n* = 13 and *n* = 1 for the patient groups with dementia and with both comorbidities, respectively). Similarly, the presence of the comorbidities was associated with greater long-term mortality risks, but no interactive effect was seen. Analysis of short-term outcomes found that patients with dementia were more likely to have a longer LoS, despite the status of diabetes. Furthermore, having dementia but not diabetes predicted membership of the third $\Delta$ mRS tertile, suggesting a possible association between isolated dementia and higher excess disability after stroke. Additionally, none of the comorbidity groups were associated with increases in in-hospital mortality risk.

With regards to isolated co-morbid diabetes, this study contributes to a knowledge pool characterizing the relationship between diabetes and long-term stroke recurrence. A US-based study previously found that while diabetes was associated with short-term and long-term mortality following stroke, it was not a significant predictor of 1-year recurrence [1]. However, a meta-analysis of studies from diverse backgrounds, found that diabetes was an independent predictor of increased stroke recurrence risk [2]. Several other cohort studies also demonstrated that patients with diabetes had a significantly higher risk of recurrence after stroke compared to those without [3, 22]. Moreover, one of these studies (investigating a Thai population) also found that patients with pre-existing diabetes had a 54% increase in long-term mortality following ischemic stroke [3]. Although the risk is greater than our study’s findings, the results together suggest an association of diabetes with long-term mortality in stroke patients. Additionally, the study of the Thai population showed an increased risk of in-hospital mortality in patients with diabetes [3] while our study and Braun et al [23] did not find this significant association. Such a disparity may be attributed to differences in ethnicity of the respective sample populations and calls for further research in this area.

Regarding post-stroke mortality risk in ischemic stroke patients with isolated dementia, two studies from 2003 and 2012 did not find an independent association [17, 24] while more recent studies have found dementia to be a significant independent predictor of long-term mortality [25, 26]. In addition to the differences in thrombolytic therapy administration rates, previous literature has also shown hesitancy from physicians in prescribing stroke prevention medications to patients with dementia [27]. Although differences in administration of antithrombotic medications were not seen in this study (possibly due to missing data), the findings in other literature suggest this may play a role in negative stroke outcomes associated with the presence of pre-existing dementia. Furthermore, reduced compliance to medications by patients with dementia may also contribute to poorer outcomes. A study assessing cognitive function and compliance to antihypertensive drugs found increased risks of non-compliance in subjects with cognitive impairment. It also showed that for people living alone, cognitive function was an independent predictor of non-compliance to the medications [28]. From a biological standpoint, there are numerous pathophysiological explanations linking different types of dementia and CVD such as hypertension, cholesterol metabolism, and diabetes [29].

This study is characterized by several strengths. First and foremost, the robust methods of data collection enabled us to use a large cohort with long-term follow-up. Due to the record linkage within the NHS, we were also able to ensure that comorbidity data were ascertained throughout the follow-up period. In our study design, many variables that were deemed relevant based on previous literature were also adjusted for. Therefore, we were able to investigate the independent impacts of the two conditions. Lastly, the stratification of patients into four mutually exclusive groups enabled us to extract findings not only on the isolated effects of each condition, but also the combined impact of the two. The inclusion of diabetes and dementia as interaction terms in the respective models also allowed us to explore any synergistic effects on stroke outcomes. These analyses together yielded novel findings which had previously not been studied prior.

This study is also subject to some limitations. As a hospital-based study, we were not able to assess the link between the co-morbidities and stroke outcomes for cases who were not admitted to the hospital. However, those not admitted to the hospitals in the UK are likely those who have had very mild cases and/or have died prior to admission. Therefore, this limitation is unlikely to have had an impact on the magnitude and direction of the effects observed. Moreover, although delirium is different from dementia, which is chronic in nature, the former was included as a diagnostic code under the dementia category. This is a result of the coding system that is followed by the NNSTR, which groups delirium alongside other types of dementia such as unspecified dementia and dementia in other diseases. Therefore, we were unable to exclude delirium in our analyses. However, delirium detection rates were low in practice during this time and therefore, this group is likely to consist of other dementias such as frontotemporal or Lewy body dementia.

Additionally, a considerable proportion of NIHSS data were missing (therefore were imputed), as data on this variable was only routinely collected from 2015 onwards. However, our sensitivity analyses showed that there were no significant differences in results whether or not the models adjusted for NIHSS. Our models also took into account of other variables related to severity of stroke such as the OCSP classifications and mRS scores. Variables that we were unable to adjust for, due to limited data availability, were family history and lifestyle factors such as exercise, smoking, alcohol, which may also play a role in long-term outcomes.

Furthermore, of note, the mean age of our sample was 80 and the mean age for the dementia group was 86, which is a relatively advanced age group. Although the registry reflects a real-world stroke population in the UK, it is important to keep in mind that sample populations of a younger age group may yield different results due to factors such as access to healthcare. It can also be noted that the prevalence of dementia in the study cohort is < 4%, which is low in a population that has a mean age of 80. This is likely to be a result of misclassification of those with dementia. This misclassification bias could have had an attenuating effect on the observed impact on stroke outcomes that the patient groups with pre-existing dementia had.

Additionally, the exposure groups were unequal in size given the nature of this study. Two of the groups, composed of patients with isolated dementia and with both comorbidities, had small sample sizes. This, alongside the subsequent low effect size, may have led to the false negative results for our short-term outcomes as seen in the results of Table 2. A greater sample size would allow us to address these limitations. It would also have allowed stratified analyses of different types of dementia, which would enable us to explore varying pathophysiology that may be associated with negative stroke outcomes. As such, we would recommend further research investigating the outcomes associated with these subtypes. Future studies should also aim to replicate our analyses on larger pools of consecutive patients from multiple centers. Furthermore, it would be of interest to explore ethnically heterogeneous patient populations as such differences have been seen in both incidence and severity of diabetes and dementia [30, 31].

Building off of previous studies, the results of this study have several implications. The vast amount of evidence on individual impacts of these conditions, combined with novel findings from our study showing the lack of a synergistic impact on stroke outcomes, suggests that despite recent studies suggesting potentially shared mechanisms between diabetes and dementia, these may not contribute to negative stroke outcomes associated with each comorbidity. Given that CVD is the leading cause of death across the globe [32], it is important that we address risk factors that may be associated with the individual pre-existing conditions that have a negative impact on stroke outcomes. Implementing preventative strategies tailored towards stroke patients with diabetes and dementia to minimize recurrence and mortality risks would be a crucial step in doing so. Further research addressing the existing controversies around care for these vulnerable groups would also be vital in guiding clinical decisions for these patients.

## Conclusion

This study confirms the independent negative impacts of pre-existing diabetes and dementia on ischemic stroke outcomes. Our study also shows that despite substantial cumulative impacts on long-term outcomes, there is no significant synergistic effects of these comorbidities on the stroke outcomes. In addressing the individual impacts on stroke recurrence, more intensive secondary prevention, such as care strategies that optimize management of the specific patient groups, are recommended. Assessment of current treatment guidance for these patient groups would also be beneficial towards implementing more specific and robust guidelines for these vulnerable patients. Doing so would have potential secondary benefits by reducing the risk of other negative outcomes such as long-term mortality. Addressing these will be a crucial step towards reducing the global CVD burden.

### Supplementary Information

Additional file 1.

Additional file 2.

Additional file 3.

Additional file 4.

Additional file 5.

Additional file 6.

Additional file 7.

## Data Availability

The datasets used during the current study are available from the corresponding author on reasonable request.

## Abbreviations

AIS: Acute Ischemic Stroke
LoS: Length of Stay
TIA: Transient Ischemic Attack
NNSTR: Norfolk and Norwich Stroke and TIA Register
ICD-10: Tenth Revision of the International Classification of Disease
OCSP: Oxfordshire Community Stroke Project classification
mRS: Modified Rankin Scale
NIHSS: National Institutes of Health Stroke Scale
IQR: Interquartile Range
CVD: Cardiovascular Disease
rt-PA: Recombinant Tissue Plasminogen Activator
