# Supplementary material for: The individual and combined impacts of pre-existing diabetes and dementia on ischemic stroke outcomes: a registry-based cohort study
Source: BMC Cardiovasc Disord. 2024 Jul 30;24:396. doi: 10.1186/s12872-024-04050-3 (PMC11290225; doi:10.1186/s12872-024-04050-3)
Supplement: Supplementary file 1 — Additional file 1. [file 12872_2024_4050_MOESM1_ESM.pdf]

Additional file 1 International Classification of Disease-10 (ICD-10) codes of confounding comorbidities

Pneumonia	J12 – J18, J69
Asthma	J45
Chronic Obstructive Pulmonary Disease	J40 – J44, J47
Transient Ischemic Attack	G45
Myocardial Infarction	I21
Hyperlipidemia	E78
Peripheral Vascular Disease	I73.9
Heart Failure	I50
Atrial Fibrillation and Flutter	I48
Hypertension	I10 – I15
Cancers	C00 – C97
Chronic Kidney Disease	N18
Liver Disease	K70 – K77
Hemorrhagic Stroke	I60, I61
Other types of stroke	I64
