# Supplementary material for: The individual and combined impacts of pre-existing diabetes and dementia on ischemic stroke outcomes: a registry-based cohort study
Source: BMC Cardiovasc Disord. 2024 Jul 30;24:396. doi: 10.1186/s12872-024-04050-3 (PMC11290225; doi:10.1186/s12872-024-04050-3)
Supplement: Supplementary file 2 — Additional file 2. [file 12872_2024_4050_MOESM2_ESM.pdf]

Additional file 2 Frequencies and percentages of missing data

Variable	Missing	
	N	%
OCSP classification	704	7
Random plasma glucose on admission	2603	24
Fasting glucose on admission	9424	87
Creatinine on admission	297	3
Sodium on admission	309	3
Albumin on admission	467	4
Cholesterol on admission	3846	36
INR on admission	1554	14
CRP on admission	1872	17
Hemoglobin on admission	556	5
White cell count on admission	268	2
Platelet count on admission	283	3
mRS before stroke	607	6
mRS after stroke	3157	29
NIHSS	9290	86
