# Supplementary material for: The individual and combined impacts of pre-existing diabetes and dementia on ischemic stroke outcomes: a registry-based cohort study
Source: BMC Cardiovasc Disord. 2024 Jul 30;24:396. doi: 10.1186/s12872-024-04050-3 (PMC11290225; doi:10.1186/s12872-024-04050-3)
Supplement: Supplementary file 3 — Additional file 3. [file 12872_2024_4050_MOESM3_ESM.pdf]

Additional file 3: Stratified tables comparing patients with missing data to those without missing data

Additional table 1 Patient characteristics stratified by whether or not data for **random plasma glucose** levels were missing

	Total	Missing	Non-missing	p-value
Age, median (IQR)	80.00 (71.63-86.00)	81.00 (72.00-87.00)	80.00 (71.00-86.00)	0.019
Sex (% female)	5623 (52.01)	1391 (53.44)	4232 (51.55)	0.093
Length of stay, median (IQR)	8.00 (3.00-17.00)	7.00 (3.00-16.00)	8.00 (3.43-17.00)	**<0.001**
mRS admission, median (IQR)	0.00 (0.00-2.00)	0.00 (0.00-2.00)	0.00 (0.00-1.00)	0.081
mRS discharge, median (IQR)	3.00 (1.00-6.00)	3.00 (1.00-6.00)	3.00 (1.00-5.00)	**<0.001**
ΔmRS, median (IQR)	2.00 (0.00-3.00)	2.00 (1.00-4.00)	2.00 (0.00-3.00)	0.004
Charlson comorbidity index, median (IQR)	3.00 (1.00-4.00)	2.00 (1.00-4.00)	3.00 (1.00-4.00)	**<0.001**
Total NIHSS, median (IQR)	4.00 (1.00-10.00)	2.00 (1.00-7.00)	4.00 (1.00-10.00)	**<0.001**
In-hospital mortality, n (%)	2042 (18.89)	633 (24.32)	1409 (17.16)	**<0.001**
Antiplatelets at admission, n (%)	3610 (33.39)	948 (36.42)	2662 (32.43)	**<0.001**
Antiplatelets at discharge, n (%)	7045 (65.16)	1609 (61.81)	5436 (66.22)	**<0.001**
Anticoagulants at admission, n (%)	169 (1.56)	38 (1.46)	131 (1.60)	0.626
Anticoagulants at discharge, n (%)	434 (4.01)	89 (3.42)	345 (4.20)	0.076
Pneumonia, n (%)	2000 (18.50)	475 (18.25)	1525 (18.58)	0.706
Asthma, n (%)	1011 (9.35)	255 (9.80)	756 (9.21)	0.370
Chronic obstructive pulmonary disease, n (%)	897 (8.30)	229 (8.80)	668 (8.14)	0.287
Transient ischemic attack, n (%)	559 (5.17)	116 (4.46)	443 (5.40)	0.059
Myocardial Infarction, n (%)	811 (7.50)	181 (6.95)	630 (7.67)	0.224
Hyperlipidemia, n (%)	1469 (13.59)	309 (11.87)	1160 (14.13)	0.003
Congenital heart disease, n (%)	3004 (27.78)	724 (27.81)	2280 (27.77)	0.969
Peripheral vascular disease, n (%)	452 (4.18)	108 (4.15)	344 (4.19)	0.927
Heart failure, n (%)	1543 (14.27)	382 (14.68)	1161 (14.14)	0.499
Atrial fibrillation, n (%)	3537 (32.71)	817 (31.39)	2720 (33.13)	0.098

Hypertension, n (%)	6592 (60.97)	1504 (57.78)	5088 (61.98)	**<0.001**
Cancers, n (%)	1771 (16.38)	417 (16.02)	1354 (16.49)	0.569
Chronic kidney disease, n (%)	719 (6.65)	161 (6.19)	558 (6.80)	0.275
Liver disease, n (%)	170 (1.57)	40 (1.54)	130 (1.58)	0.867
Random plasma glucose, median (IQR)	-	-	-	-
Fasting glucose, median (IQR)	5.50 (5.00-6.40)	5.60 (5.00-6.40)	5.50 (5.00-6.40)	0.320
Creatinine, median (IQR)	86.00 (71.00-108.00)	87.00 (72.00-110.00)	86.00 (71.00-108.00)	0.039
Sodium, mean (SD)	138.30 (4.23)	138.46 (4.41)	138.26 (4.18)	0.050
Albumin, mean (SD)	36.63 (5.43)	35.97 (6.13)	36.82 (5.20)	**<0.001**
Cholesterol, mean (SD)	4.85 (1.31)	4.84 (1.29)	4.85 (1.31)	0.656
INR, median (IQR)	1.04 (0.98-1.13)	1.04 (0.98-1.14)	1.04 (0.98-1.12)	0.339
CRP, median (IQR)	11.00 (5.00-36.00)	14.00 (5.00-44.00)	11.00 (5.00-33.00)	**<0.001**
Hemoglobin, mean (SD)	134.61 (19.57)	132.54 (20.73)	135.19 (19.20)	**<0.001**
White cell count, median (IQR)	8.80 (7.10-11.30)	8.90 (7.10-11.90)	8.80 (7.10-11.20)	0.004
Platelet count, median (IQR)	245.00 (201.00-302.00)	248.00 (200.00-309.00)	244.00 (201.00-300.00)	0.091

Additional table 2 Patient characteristics stratified by whether or not data for **fasting glucose** levels were missing

	Total	Missing	Non-missing	p-value
Age, median (IQR)	80.00 (71.63-86.00)	81.00 (72.00-87.00)	78.00 (68.00-84.00)	**<0.001**
Sex (% female)	5623 (52.01)	4941 (52.43)	682 (49.14)	0.022
Length of stay, median (IQR)	8.00 (3.00-17.00)	8.00 (3.00-17.00)	8.00 (4.00-17.00)	0.092
mRS admission, median (IQR)	0.00 (0.00-2.00)	0.00 (0.00-2.00)	0.00 (0.00-1.00)	**<0.001**
mRS discharge, median (IQR)	3.00 (1.00-6.00)	3.00 (1.00-6.00)	2.00 (1.00-4.00)	**<0.001**
ΔmRS, median (IQR)	2.00 (0.00-3.00)	2.00 (0.00-3.00)	1.00 (0.00-3.00)	**<0.001**
Charlson comorbidity index, median (IQR)	3.00 (1.00-4.00)	3.00 (1.00-4.00)	3.00 (1.00-4.00)	0.554
Total NIHSS, median (IQR)	4.00 (1.00-10.00)	4.00 (1.00-10.00)	3.50 (0.00-15.00)	0.890
In-hospital mortality, n (%)	2042 (18.89)	1931 (20.49)	111 (8.00)	**<0.001**
Antiplatelets at admission, n (%)	3610 (33.39)	3141 (33.33)	469 (33.79)	0.735
Antiplatelets at discharge, n (%)	7045 (65.16)	5929 (62.91)	1116 (80.40)	**<0.001**
Anticoagulants at admission, n (%)	169 (1.56)	165 (1.75)	4 (0.29)	**<0.001**
Anticoagulants at discharge, n (%)	434 (4.01)	421 (4.47)	13 (0.94)	**<0.001**
Pneumonia, n (%)	2000 (18.50)	1834 (19.46)	166 (11.96)	**<0.001**
Asthma, n (%)	1011 (9.35)	882 (9.36)	129 (9.29)	0.938
Chronic obstructive pulmonary disease, n (%)	897 (8.30)	802 (8.51)	95 (6.84)	0.036
Transient ischemic attack, n (%)	559 (5.17)	495 (5.25)	64 (4.61)	0.314
Myocardial Infarction, n (%)	811 (7.50)	717 (7.61)	94 (6.77)	0.270
Hyperlipidemia, n (%)	1469 (13.59)	1235 (13.10)	234 (16.86)	**<0.001**
Congenital heart disease, n (%)	3004 (27.78)	2668 (28.31)	336 (24.21)	0.001
Peripheral vascular disease, n (%)	452 (4.18)	393 (4.17)	59 (4.25)	0.889
Heart failure, n (%)	1543 (14.27)	1390 (14.75)	153 (11.02)	**<0.001**
Atrial fibrillation, n (%)	3537 (32.71)	3132 (33.23)	405 (29.18)	0.003
Hypertension, n (%)	6592 (60.97)	5742 (60.93)	850 (61.24)	0.825
Cancers, n (%)	1771 (16.38)	1567 (16.63)	204 (14.70)	0.070

Chronic kidney disease, n (%)	719 (6.65)	655 (6.95)	64 (4.61)	0.001
Liver disease, n (%)	170 (1.57)	159 (1.69)	11 (0.79)	0.012
Random plasma glucose, median (IQR)	6.30 (5.50-7.80)	6.30 (5.50-7.80)	6.40 (5.60-8.20)	0.068
Fasting glucose, median (IQR)	-	-	-	-
Creatinine, median (IQR)	86.00 (71.00-108.00)	86.00 (71.00-109.00)	83.00 (71.00-100.00)	**<0.001**
Sodium, mean (SD)	138.30 (4.23)	138.27 (4.29)	138.53 (3.84)	0.030
Albumin, mean (SD)	36.63 (5.43)	36.46 (5.52)	37.74 (4.60)	**<0.001**
Cholesterol, mean (SD)	4.85 (1.31)	4.82 (1.30)	4.98 (1.33)	**<0.001**
INR, median (IQR)	1.04 (0.98-1.13)	1.04 (0.98-1.13)	1.03 (0.97-1.09)	**<0.001**
CRP, median (IQR)	11.00 (5.00-36.00)	12.00 (5.00-38.00)	10.00 (5.00-25.00)	0.001
Hemoglobin, mean (SD)	134.61 (19.57)	134.18 (19.71)	137.43 (18.40)	**<0.001**
White cell count, median (IQR)	8.80 (7.10-11.30)	8.80 (7.20-11.30)	8.60 (7.00-11.00)	0.005
Platelet count, median (IQR)	245.00 (201.00-302.00)	245.00 (200.00-302.00)	247.00 (203.00-299.00)	0.613

Additional table 3 Patient characteristics stratified by whether or not data for **creatinine** levels were missing

	Total	Missing	Non-missing	p-value
Age, median (IQR)	80.00 (71.63-86.00)	80.00 (71.00-86.00)	80.00 (71.67-86.00)	0.388
Sex (% female)	5623 (52.01)	137 (46.13)	5486 (52.17)	0.040
Length of stay, median (IQR)	8.00 (3.00-17.00)	5.00 (2.00-12.00)	8.00 (3.16-17.00)	**<0.001**
mRS admission, median (IQR)	0.00 (0.00-2.00)	0.00 (0.00-1.00)	0.00 (0.00-2.00)	0.086
mRS discharge, median (IQR)	3.00 (1.00-6.00)	2.00 (0.00-6.00)	3.00 (1.00-6.00)	**<0.001**
ΔmRS, median (IQR)	2.00 (0.00-3.00)	1.00 (0.00-3.00)	2.00 (0.00-3.00)	0.001
Charlson comorbidity index, median (IQR)	3.00 (1.00-4.00)	2.00 (1.00-3.00)	3.00 (1.00-4.00)	**<0.001**
Total NIHSS, median (IQR)	4.00 (1.00-10.00)	18.00 (5.50-25.50)	4.00 (1.00-10.00)	0.172
In-hospital mortality, n (%)	2042 (18.89)	61 (20.54)	1981 (18.84)	0.461
Antiplatelets at admission, n (%)	3610 (33.39)	83 (27.95)	3527 (33.54)	0.044
Antiplatelets at discharge, n (%)	7045 (65.16)	150 (50.51)	6895 (65.57)	**<0.001**
Anticoagulants at admission, n (%)	169 (1.56)	3 (1.01)	166 (1.58)	0.436
Anticoagulants at discharge, n (%)	434 (4.01)	12 (4.04)	422 (4.01)	0.981
Pneumonia, n (%)	2000 (18.50)	26 (8.75)	1974 (18.77)	**<0.001**
Asthma, n (%)	1011 (9.35)	27 (9.09)	984 (9.36)	0.876
Chronic obstructive pulmonary disease, n (%)	897 (8.30)	13 (4.38)	884 (8.41)	0.013
Transient ischemic attack, n (%)	559 (5.17)	12 (4.04)	547 (5.20)	0.373
Myocardial Infarction, n (%)	811 (7.50)	12 (4.04)	799 (7.60)	0.022
Hyperlipidemia, n (%)	1469 (13.59)	42 (14.14)	1427 (13.57)	0.777
Congenital heart disease, n (%)	3004 (27.78)	46 (15.49)	2958 (28.13)	**<0.001**
Peripheral vascular disease, n (%)	452 (4.18)	11 (3.70)	441 (4.19)	0.677
Heart failure, n (%)	1543 (14.27)	15 (5.05)	1528 (14.53)	**<0.001**
Atrial fibrillation, n (%)	3537 (32.71)	58 (19.53)	3479 (33.09)	**<0.001**
Hypertension, n (%)	6592 (60.97)	132 (44.44)	6460 (61.44)	**<0.001**

Cancers, n (%)	1771 (16.38)	41 (13.80)	1730 (16.45)	0.224
Chronic kidney disease, n (%)	719 (6.65)	16 (5.39)	703 (6.69)	0.376
Liver disease, n (%)	170 (1.57)	2 (0.67)	168 (1.60)	0.207
Random plasma glucose, median (IQR)	6.30 (5.50-7.80)	5.80 (5.45-6.10)	6.30 (5.50-7.80)	0.260
Fasting glucose, median (IQR)	5.50 (5.00-6.40)	5.40 (5.20-6.80)	5.50 (5.00-6.40)	0.995
Creatinine, median (IQR)	-	-	-	-
Sodium, mean (SD)	138.30 (4.23)	132.67 (2.52)	138.30 (4.23)	0.021
Albumin, mean (SD)	36.63 (5.43)	37.94 (2.84)	36.62 (5.44)	0.084
Cholesterol, mean (SD)	4.85 (1.31)	5.23 (1.26)	4.85 (1.31)	0.064
INR, median (IQR)	1.04 (0.98-1.13)	0.97 (0.94-1.02)	1.04 (0.98-1.13)	**<0.001**
CRP, median (IQR)	11.00 (5.00-36.00)	4.00 (3.00-9.00)	11.00 (5.00-36.00)	**<0.001**
Hemoglobin, mean (SD)	134.61 (19.57)	138.03 (17.57)	134.60 (19.58)	0.337
White cell count, median (IQR)	8.80 (7.10-11.30)	8.20 (6.80-9.90)	8.80 (7.10-11.30)	0.037
Platelet count, median (IQR)	245.00 (201.00-302.00)	265.00 (232.00-309.00)	245.00 (200.00-302.00)	0.065

Additional table 4 Patient characteristics stratified by whether or not data for **albumin** levels were missing

	Total	Missing	Non-missing	p-value
Age, median (IQR)	80.00 (71.63-86.00)	80.00 (71.00-86.00)	80.00 (71.82-86.00)	0.249
Sex (% female)	5623 (52.01)	220 (47.11)	5403 (52.23)	0.030
Length of stay, median (IQR)	8.00 (3.00-17.00)	5.00 (2.00-11.00)	8.00 (3.22-17.00)	**<0.001**
mRS admission, median (IQR)	0.00 (0.00-2.00)	0.00 (0.00-1.00)	0.00 (0.00-2.00)	0.006
mRS discharge, median (IQR)	3.00 (1.00-6.00)	3.00 (1.00-6.00)	3.00 (1.00-6.00)	0.975
ΔmRS, median (IQR)	2.00 (0.00-3.00)	1.00 (0.00-4.00)	2.00 (0.00-3.00)	0.410
Charlson comorbidity index, median (IQR)	3.00 (1.00-4.00)	2.00 (1.00-4.00)	3.00 (1.00-4.00)	**<0.001**
Total NIHSS, median (IQR)	4.00 (1.00-10.00)	5.00 (1.00-11.00)	4.00 (1.00-10.00)	0.927
In-hospital mortality, n (%)	2042 (18.89)	117 (25.05)	1925 (18.61)	**<0.001**
Antiplatelets at admission, n (%)	3610 (33.39)	150 (32.12)	3460 (33.45)	0.552
Antiplatelets at discharge, n (%)	7045 (65.16)	231 (49.46)	6814 (65.87)	**<0.001**
Anticoagulants at admission, n (%)	169 (1.56)	4 (0.86)	165 (1.59)	0.208
Anticoagulants at discharge, n (%)	434 (4.01)	16 (3.43)	418 (4.04)	0.508
Pneumonia, n (%)	2000 (18.50)	51 (10.92)	1949 (18.84)	**<0.001**
Asthma, n (%)	1011 (9.35)	38 (8.14)	973 (9.41)	0.357
Chronic obstructive pulmonary disease, n (%)	897 (8.30)	31 (6.64)	866 (8.37)	0.184
Transient ischemic attack, n (%)	559 (5.17)	17 (3.64)	542 (5.24)	0.127
Myocardial Infarction, n (%)	811 (7.50)	20 (4.28)	791 (7.65)	0.007
Hyperlipidemia, n (%)	1469 (13.59)	56 (11.99)	1413 (13.66)	0.304
Congenital heart disease, n (%)	3004 (27.78)	91 (19.49)	2913 (28.16)	**<0.001**
Peripheral vascular disease, n (%)	452 (4.18)	15 (3.21)	437 (4.22)	0.285
Heart failure, n (%)	1543 (14.27)	38 (8.14)	1505 (14.55)	**<0.001**
Atrial fibrillation, n (%)	3537 (32.71)	102 (21.84)	3435 (33.20)	**<0.001**
Hypertension, n (%)	6592 (60.97)	232 (49.68)	6360 (61.48)	**<0.001**

Cancers, n (%)	1771 (16.38)	70 (14.99)	1701 (16.44)	0.406
Chronic kidney disease, n (%)	719 (6.65)	23 (4.93)	696 (6.73)	0.126
Liver disease, n (%)	170 (1.57)	3 (0.64)	167 (1.61)	0.099
Random plasma glucose, median (IQR)	6.30 (5.50-7.80)	6.40 (5.60-8.40)	6.30 (5.50-7.80)	0.451
Fasting glucose, median (IQR)	5.50 (5.00-6.40)	5.60 (5.10-7.10)	5.50 (5.00-6.40)	0.381
Creatinine, median (IQR)	86.00 (71.00-108.00)	82.00 (71.00-102.00)	86.00 (71.00-108.00)	0.190
Sodium, mean (SD)	138.30 (4.23)	138.95 (3.58)	138.29 (4.25)	0.025
Albumin, mean (SD)	-	-	-	**-**
Cholesterol, mean (SD)	4.85 (1.31)	4.95 (1.18)	4.85 (1.31)	0.455
INR, median (IQR)	1.04 (0.98-1.13)	1.02 (0.95-1.08)	1.04 (0.98-1.13)	**<0.001**
CRP, median (IQR)	11.00 (5.00-36.00)	8.00 (4.00-30.50)	11.00 (5.00-36.00)	0.064
Hemoglobin, mean (SD)	134.61 (19.57)	137.31 (19.17)	134.55 (19.58)	0.042
White cell count, median (IQR)	8.80 (7.10-11.30)	8.80 (7.00-11.50)	8.80 (7.10-11.30)	0.918
Platelet count, median (IQR)	245.00 (201.00-302.00)	244.00 (202.00-303.00)	245.00 (201.00-302.00)	0.853

Additional table 5 Patient characteristics stratified by whether or not data for **sodium** levels were missing

	Total	Missing	Non-missing	p-value
Age, median (IQR)	80.00 (71.63-86.00)	80.00 (70.00-86.00)	80.00 (71.75-86.00)	0.306
Sex (% female)	5623 (52.01)	143 (46.28)	5480 (52.18)	0.041
Length of stay, median (IQR)	8.00 (3.00-17.00)	5.00 (2.00-11.00)	8.00 (3.17-17.00)	**<0.001**
mRS admission, median (IQR)	0.00 (0.00-2.00)	0.00 (0.00-1.00)	0.00 (0.00-2.00)	0.080
mRS discharge, median (IQR)	3.00 (1.00-6.00)	2.00 (0.00-6.00)	3.00 (1.00-6.00)	0.004
ΔmRS, median (IQR)	2.00 (0.00-3.00)	1.00 (0.00-3.00)	2.00 (0.00-3.00)	0.009
Charlson comorbidity index, median (IQR)	3.00 (1.00-4.00)	2.00 (1.00-3.00)	3.00 (1.00-4.00)	**<0.001**
Total NIHSS, median (IQR)	4.00 (1.00-10.00)	25.00 (0.00-26.00)	4.00 (1.00-10.00)	0.308
In-hospital mortality, n (%)	2042 (18.89)	68 (22.01)	1974 (18.79)	0.155
Antiplatelets at admission, n (%)	3610 (33.39)	87 (28.16)	3523 (33.54)	0.048
Antiplatelets at discharge, n (%)	7045 (65.16)	152 (49.19)	6893 (65.63)	**<0.001**
Anticoagulants at admission, n (%)	169 (1.56)	3 (0.97)	166 (1.58)	0.394
Anticoagulants at discharge, n (%)	434 (4.01)	12 (3.88)	422 (4.02)	0.906
Pneumonia, n (%)	2000 (18.50)	28 (9.06)	1972 (18.78)	**<0.001**
Asthma, n (%)	1011 (9.35)	28 (9.06)	983 (9.36)	0.859
Chronic obstructive pulmonary disease, n (%)	897 (8.30)	13 (4.21)	884 (8.42)	0.008
Transient ischemic attack, n (%)	559 (5.17)	13 (4.21)	546 (5.20)	0.438
Myocardial Infarction, n (%)	811 (7.50)	14 (4.53)	797 (7.59)	0.044
Hyperlipidemia, n (%)	1469 (13.59)	41 (13.27)	1428 (13.60)	0.868
Congenital heart disease, n (%)	3004 (27.78)	48 (15.53)	2956 (28.14)	**<0.001**
Peripheral vascular disease, n (%)	452 (4.18)	11 (3.56)	441 (4.20)	0.580
Heart failure, n (%)	1543 (14.27)	19 (6.15)	1524 (14.51)	**<0.001**
Atrial fibrillation, n (%)	3537 (32.71)	60 (19.42)	3477 (33.10)	**<0.001**
Hypertension, n (%)	6592 (60.97)	137 (44.34)	6455 (61.46)	**<0.001**

Cancers, n (%)	1771 (16.38)	41 (13.27)	1730 (16.47)	0.134
Chronic kidney disease, n (%)	719 (6.65)	17 (5.50)	702 (6.68)	0.411
Liver disease, n (%)	170 (1.57)	2 (0.65)	168 (1.60)	0.185
Random plasma glucose, median (IQR)	6.30 (5.50-7.80)	6.20 (5.30-10.10)	6.30 (5.50-7.80)	0.765
Fasting glucose, median (IQR)	5.50 (5.00-6.40)	5.50 (5.20-6.80)	5.50 (5.00-6.40)	0.756
Creatinine, median (IQR)	86.00 (71.00-108.00)	75.00 (53.00-94.00)	86.00 (71.00-108.00)	0.080
Sodium, mean (SD)	-	-	-	-
Albumin, mean (SD)	36.63 (5.43)	38.31 (3.38)	36.62 (5.43)	0.026
Cholesterol, mean (SD)	4.85 (1.31)	5.24 (1.21)	4.85 (1.31)	0.041
INR, median (IQR)	1.04 (0.98-1.13)	0.97 (0.92-1.02)	1.04 (0.98-1.13)	**<0.001**
CRP, median (IQR)	11.00 (5.00-36.00)	4.00 (3.00-9.00)	11.00 (5.00-36.00)	**<0.001**
Hemoglobin, mean (SD)	134.61 (19.57)	140.79 (15.85)	134.58 (19.58)	0.040
White cell count, median (IQR)	8.80 (7.10-11.30)	8.30 (6.80-10.90)	8.80 (7.10-11.30)	0.125
Platelet count, median (IQR)	245.00 (201.00-302.00)	265.00 (226.00-295.00)	245.00 (200.00-302.00)	0.182

Additional table 6 Patient characteristics stratified by whether or not data for **cholesterol** levels were missing

	Total	Missing	Non-missing	p-value
Age, median (IQR)	80.00 (71.63-86.00)	82.00 (74.00-88.00)	79.00 (70.00-85.35)	**<0.001**
Sex (% female)	5623 (52.01)	2127 (55.30)	3496 (50.19)	**<0.001**
Length of stay, median (IQR)	8.00 (3.00-17.00)	7.00 (3.00-16.00)	8.00 (4.00-18.00)	**<0.001**
mRS admission, median (IQR)	0.00 (0.00-2.00)	0.00 (0.00-2.00)	0.00 (0.00-1.00)	**<0.001**
mRS discharge, median (IQR)	3.00 (1.00-6.00)	4.00 (2.00-6.00)	3.00 (1.00-4.00)	**<0.001**
ΔmRS, median (IQR)	2.00 (0.00-3.00)	2.00 (1.00-4.00)	2.00 (0.00-3.00)	**<0.001**
Charlson comorbidity index, median (IQR)	3.00 (1.00-4.00)	3.00 (1.00-4.00)	3.00 (1.00-4.00)	0.865
Total NIHSS, median (IQR)	4.00 (1.00-10.00)	2.00 (0.00-8.00)	4.00 (2.00-10.00)	**<0.001**
In-hospital mortality, n (%)	2042 (18.89)	1222 (31.77)	820 (11.77)	**<0.001**
Antiplatelets at admission, n (%)	3610 (33.39)	1493 (38.82)	2117 (30.39)	**<0.001**
Antiplatelets at discharge, n (%)	7045 (65.16)	2044 (53.15)	5001 (71.79)	**<0.001**
Anticoagulants at admission, n (%)	169 (1.56)	33 (0.86)	136 (1.95)	**<0.001**
Anticoagulants at discharge, n (%)	434 (4.01)	99 (2.57)	335 (4.81)	**<0.001**
Pneumonia, n (%)	2000 (18.50)	881 (22.91)	1119 (16.06)	**<0.001**
Asthma, n (%)	1011 (9.35)	365 (9.49)	646 (9.27)	0.711
Chronic obstructive pulmonary disease, n (%)	897 (8.30)	338 (8.79)	559 (8.02)	0.168
Transient ischemic attack, n (%)	559 (5.17)	173 (4.50)	386 (5.54)	0.019
Myocardial Infarction, n (%)	811 (7.50)	308 (8.01)	503 (7.22)	0.137
Hyperlipidemia, n (%)	1469 (13.59)	408 (10.61)	1061 (15.23)	**<0.001**
Congenital heart disease, n (%)	3004 (27.78)	1136 (29.54)	1868 (26.82)	0.002
Peripheral vascular disease, n (%)	452 (4.18)	177 (4.60)	275 (3.95)	0.104
Heart failure, n (%)	1543 (14.27)	622 (16.17)	921 (13.22)	**<0.001**
Atrial fibrillation, n (%)	3537 (32.71)	1361 (35.39)	2176 (31.24)	**<0.001**
Hypertension, n (%)	6592 (60.97)	2282 (59.33)	4310 (61.87)	0.010

Cancers, n (%)	1771 (16.38)	688 (17.89)	1083 (15.55)	0.002
Chronic kidney disease, n (%)	719 (6.65)	237 (6.16)	482 (6.92)	0.130
Liver disease, n (%)	170 (1.57)	61 (1.59)	109 (1.56)	0.932
Random plasma glucose, median (IQR)	6.30 (5.50-7.80)	6.50 (5.60-8.30)	6.30 (5.50-7.70)	**<0.001**
Fasting glucose, median (IQR)	5.50 (5.00-6.40)	6.20 (5.40-7.20)	5.50 (5.00-6.40)	0.004
Creatinine, median (IQR)	86.00 (71.00-108.00)	87.00 (71.00-114.00)	85.00 (71.00-106.00)	**<0.001**
Sodium, mean (SD)	138.30 (4.23)	138.17 (4.86)	138.37 (3.87)	0.019
Albumin, mean (SD)	36.63 (5.43)	35.47 (6.13)	37.22 (4.93)	**<0.001**
Cholesterol, mean (SD)	-	-	-	-
INR, median (IQR)	1.04 (0.98-1.13)	1.06 (0.99-1.16)	1.03 (0.97-1.11)	**<0.001**
CRP, median (IQR)	11.00 (5.00-36.00)	18.00 (6.00-53.00)	10.00 (4.00-28.00)	**<0.001**
Hemoglobin, mean (SD)	134.61 (19.57)	131.62 (20.45)	136.16 (18.92)	**<0.001**
White cell count, median (IQR)	8.80 (7.10-11.30)	9.10 (7.30-12.10)	8.70 (7.10-11.00)	**<0.001**
Platelet count, median (IQR)	245.00 (201.00-302.00)	249.00 (199.00-307.00)	244.00 (201.00-300.00)	0.062

Additional table 7 Patient characteristics stratified by whether or not data for **INR** levels were missing

	Total	Missing	Non-missing	p-value
Age, median (IQR)	80.00 (71.63-86.00)	82.00 (73.00-87.00)	80.00 (71.00-86.00)	**<0.001**
Sex (% female)	5623 (52.01)	874 (56.24)	4749 (51.30)	**<0.001**
Length of stay, median (IQR)	8.00 (3.00-17.00)	7.00 (3.00-15.00)	8.00 (3.17-18.00)	**<0.001**
mRS admission, median (IQR)	0.00 (0.00-2.00)	0.00 (0.00-2.00)	0.00 (0.00-1.00)	0.032
mRS discharge, median (IQR)	3.00 (1.00-6.00)	4.00 (1.00-6.00)	3.00 (1.00-5.00)	<**0.001**
ΔmRS, median (IQR)	2.00 (0.00-3.00)	2.00 (0.50-4.00)	2.00 (0.00-3.00)	0.093
Charlson comorbidity index, median (IQR)	3.00 (1.00-4.00)	2.00 (1.00-4.00)	3.00 (1.00-4.00)	**<0.001**
Total NIHSS, median (IQR)	4.00 (1.00-10.00)	2.00 (0.00-5.00)	4.00 (1.00-10.00)	**<0.001**
In-hospital mortality, n (%)	2042 (18.89)	406 (26.13)	1636 (17.67)	**<0.001**
Antiplatelets at admission, n (%)	3610 (33.39)	488 (31.40)	3122 (33.72)	0.073
Antiplatelets at discharge, n (%)	7045 (65.16)	879 (56.56)	6166 (66.60)	**<0.001**
Anticoagulants at admission, n (%)	169 (1.56)	12 (0.77)	157 (1.70)	0.007
Anticoagulants at discharge, n (%)	434 (4.01)	41 (2.64)	393 (4.24)	0.003
Pneumonia, n (%)	2000 (18.50)	268 (17.25)	1732 (18.71)	0.169
Asthma, n (%)	1011 (9.35)	100 (6.44)	911 (9.84)	**<0.001**
Chronic obstructive pulmonary disease, n (%)	897 (8.30)	102 (6.56)	795 (8.59)	0.007
Transient ischemic attack, n (%)	559 (5.17)	75 (4.83)	484 (5.23)	0.508
Myocardial Infarction, n (%)	811 (7.50)	86 (5.53)	725 (7.83)	0.001
Hyperlipidemia, n (%)	1469 (13.59)	164 (10.55)	1305 (14.10)	**<0.001**
Congenital heart disease, n (%)	3004 (27.78)	378 (24.32)	2626 (28.36)	0.001
Peripheral vascular disease, n (%)	452 (4.18)	51 (3.28)	401 (4.33)	0.056
Heart failure, n (%)	1543 (14.27)	178 (11.45)	1365 (14.74)	**<0.001**
Atrial fibrillation, n (%)	3537 (32.71)	352 (22.65)	3185 (34.40)	**<0.001**
Hypertension, n (%)	6592 (60.97)	832 (53.54)	5760 (62.22)	**<0.001**

Cancers, n (%)	1771 (16.38)	218 (14.03)	1553 (16.77)	0.007
Chronic kidney disease, n (%)	719 (6.65)	66 (4.25)	653 (7.05)	**<0.001**
Liver disease, n (%)	170 (1.57)	14 (0.90)	156 (1.69)	0.021
Random plasma glucose, median (IQR)	6.30 (5.50-7.80)	6.40 (5.50-7.90)	6.30 (5.50-7.80)	0.557
Fasting glucose, median (IQR)	5.50 (5.00-6.40)	5.60 (5.00-6.50)	5.50 (5.00-6.40)	0.275
Creatinine, median (IQR)	86.00 (71.00-108.00)	85.00 (71.00-107.00)	86.00 (71.00-108.00)	0.541
Sodium, mean (SD)	138.30 (4.23)	138.32 (4.43)	138.30 (4.21)	0.884
Albumin, mean (SD)	36.63 (5.43)	36.59 (5.81)	36.64 (5.37)	0.776
Cholesterol, mean (SD)	4.85 (1.31)	4.89 (1.22)	4.85 (1.32)	0.392
INR, median (IQR)	-	-	-	-
CRP, median (IQR)	11.00 (5.00-36.00)	17.00 (6.00-48.00)	11.00 (5.00-35.00)	**<0.001**
Hemoglobin, mean (SD)	134.61 (19.57)	133.66 (19.59)	134.74 (19.57)	0.064
White cell count, median (IQR)	8.80 (7.10-11.30)	9.40 (7.40-11.90)	8.80 (7.10-11.20)	**<0.001**
Platelet count, median (IQR)	245.00 (201.00-302.00)	254.00 (206.00-311.00)	244.00 (199.00-300.50)	**<0.001**

Additional table 8 Patient characteristics stratified by whether or not data for **CRP** levels were missing

	Total	Missing	Non-missing	p-value
Age, median (IQR)	80.00 (71.63-86.00)	77.00 (67.00-84.00)	81.00 (72.00-87.00)	**<0.001**
Sex (% female)	5623 (52.01)	919 (49.09)	4704 (52.62)	0.005
Length of stay, median (IQR)	8.00 (3.00-17.00)	5.00 (2.00-9.00)	9.00 (4.00-19.00)	**<0.001**
mRS admission, median (IQR)	0.00 (0.00-2.00)	0.00 (0.00-0.00)	0.00 (0.00-2.00)	**<0.001**
mRS discharge, median (IQR)	3.00 (1.00-6.00)	2.00 (1.00-6.00)	3.00 (1.00-6.00)	**<0.001**
ΔmRS, median (IQR)	2.00 (0.00-3.00)	1.00 (0.00-3.00)	2.00 (1.00-3.00)	**<0.001**
Charlson comorbidity index, median (IQR)	3.00 (1.00-4.00)	2.00 (1.00-4.00)	3.00 (1.00-4.00)	**<0.001**
Total NIHSS, median (IQR)	4.00 (1.00-10.00)	2.00 (1.00-4.00)	4.00 (1.00-10.00)	**<0.001**
In-hospital mortality, n (%)	2042 (18.89)	283 (15.12)	1759 (19.68)	**<0.001**
Antiplatelets at admission, n (%)	3610 (33.39)	617 (32.96)	2993 (33.48)	0.665
Antiplatelets at discharge, n (%)	7045 (65.16)	1247 (66.61)	5798 (64.85)	0.146
Anticoagulants at admission, n (%)	169 (1.56)	16 (0.85)	153 (1.71)	0.007
Anticoagulants at discharge, n (%)	434 (4.01)	37 (1.98)	397 (4.44)	**<0.001**
Pneumonia, n (%)	2000 (18.50)	140 (7.48)	1860 (20.81)	**<0.001**
Asthma, n (%)	1011 (9.35)	155 (8.28)	856 (9.57)	0.080
Chronic obstructive pulmonary disease, n (%)	897 (8.30)	96 (5.13)	801 (8.96)	**<0.001**
Transient ischemic attack, n (%)	559 (5.17)	95 (5.07)	464 (5.19)	0.838
Myocardial Infarction, n (%)	811 (7.50)	88 (4.70)	723 (8.09)	**<0.001**
Hyperlipidemia, n (%)	1469 (13.59)	262 (14.00)	1207 (13.50)	0.570
Congenital heart disease, n (%)	3004 (27.78)	407 (21.74)	2597 (29.05)	**<0.001**
Peripheral vascular disease, n (%)	452 (4.18)	42 (2.24)	410 (4.59)	**<0.001**
Heart failure, n (%)	1543 (14.27)	155 (8.28)	1388 (15.53)	**<0.001**
Atrial fibrillation, n (%)	3537 (32.71)	396 (21.15)	3141 (35.13)	**<0.001**
Hypertension, n (%)	6592 (60.97)	997 (53.26)	5595 (62.58)	**<0.001**

Cancers, n (%)	1771 (16.38)	245 (13.09)	1526 (17.07)	**<0.001**
Chronic kidney disease, n (%)	719 (6.65)	57 (3.04)	662 (7.40)	**<0.001**
Liver disease, n (%)	170 (1.57)	11 (0.59)	159 (1.78)	**<0.001**
Random plasma glucose, median (IQR)	6.30 (5.50-7.80)	6.00 (5.30-7.30)	6.40 (5.50-7.90)	**<0.001**
Fasting glucose, median (IQR)	5.50 (5.00-6.40)	5.30 (4.80-5.80)	5.60 (5.00-6.50)	**<0.001**
Creatinine, median (IQR)	86.00 (71.00-108.00)	83.00 (70.00-100.00)	87.00 (71.00-110.00)	**<0.001**
Sodium, mean (SD)	138.30 (4.23)	138.91 (3.60)	138.19 (4.33)	**<0.001**
Albumin, mean (SD)	36.63 (5.43)	39.43 (4.10)	36.15 (5.48)	**<0.001**
Cholesterol, mean (SD)	4.85 (1.31)	5.18 (1.27)	4.80 (1.31)	**<0.001**
INR, median (IQR)	1.04 (0.98-1.13)	1.01 (0.96-1.07)	1.04 (0.98-1.13)	**<0.001**
CRP, median (IQR)	-	-	-	-
Hemoglobin, mean (SD)	134.61 (19.57)	139.41 (16.11)	133.73 (20.02)	**<0.001**
White cell count, median (IQR)	8.80 (7.10-11.30)	8.00 (6.60-9.90)	9.00 (7.30-11.50)	**<0.001**
Platelet count, median (IQR)	245.00 (201.00-302.00)	239.00 (201.00-287.00)	247.00 (200.00-305.00)	**<0.001**

Additional table 9 Patient characteristics stratified by whether or not data for **Hemoglobin** levels were missing

	Total	Missing	Non-missing	p-value
Age, median (IQR)	80.00 (71.63-86.00)	80.00 (70.00-86.00)	80.00 (71.78-86.00)	0.297
Sex (% female)	5623 (52.01)	278 (50.00)	5345 (52.12)	0.331
Length of stay, median (IQR)	8.00 (3.00-17.00)	6.00 (2.59-13.00)	8.00 (3.17-17.00)	**<0.001**
mRS admission, median (IQR)	0.00 (0.00-2.00)	0.00 (0.00-1.00)	0.00 (0.00-2.00)	0.107
mRS discharge, median (IQR)	3.00 (1.00-6.00)	2.00 (1.00-4.00)	3.00 (1.00-6.00)	**<0.001**
ΔmRS, median (IQR)	2.00 (0.00-3.00)	1.00 (0.00-3.00)	2.00 (0.00-3.00)	**<0.001**
Charlson comorbidity index, median (IQR)	3.00 (1.00-4.00)	2.00 (1.00-4.00)	3.00 (1.00-4.00)	**<0.001**
Total NIHSS, median (IQR)	4.00 (1.00-10.00)	13.50 (1.00-25.50)	4.00 (1.00-10.00)	0.555
In-hospital mortality, n (%)	2042 (18.89)	105 (18.88)	1937 (18.89)	0.999
Antiplatelets at admission, n (%)	3610 (33.39)	106 (19.06)	3504 (34.17)	**<0.001**
Antiplatelets at discharge, n (%)	7045 (65.16)	349 (62.77)	6696 (65.29)	0.225
Anticoagulants at admission, n (%)	169 (1.56)	4 (0.72)	165 (1.61)	0.100
Anticoagulants at discharge, n (%)	434 (4.01)	13 (2.34)	421 (4.10)	0.039
Pneumonia, n (%)	2000 (18.50)	83 (14.93)	1917 (18.69)	0.026
Asthma, n (%)	1011 (9.35)	49 (8.81)	962 (9.38)	0.655
Chronic obstructive pulmonary disease, n (%)	897 (8.30)	36 (6.47)	861 (8.40)	0.110
Transient ischemic attack, n (%)	559 (5.17)	32 (5.76)	527 (5.14)	0.522
Myocardial Infarction, n (%)	811 (7.50)	36 (6.47)	775 (7.56)	0.346
Hyperlipidemia, n (%)	1469 (13.59)	86 (15.47)	1383 (13.48)	0.184
Congenital heart disease, n (%)	3004 (27.78)	130 (23.38)	2874 (28.02)	0.017
Peripheral vascular disease, n (%)	452 (4.18)	19 (3.42)	433 (4.22)	0.356
Heart failure, n (%)	1543 (14.27)	61 (10.97)	1482 (14.45)	0.022
Atrial fibrillation, n (%)	3537 (32.71)	160 (28.78)	3377 (32.93)	0.042
Hypertension, n (%)	6592 (60.97)	307 (55.22)	6285 (61.28)	0.004

Cancers, n (%)	1771 (16.38)	91 (16.37)	1680 (16.38)	0.993
Chronic kidney disease, n (%)	719 (6.65)	40 (7.19)	679 (6.62)	0.597
Liver disease, n (%)	170 (1.57)	9 (1.62)	161 (1.57)	0.928
Random plasma glucose, median (IQR)	6.30 (5.50-7.80)	6.40 (5.60-8.25)	6.30 (5.50-7.80)	0.395
Fasting glucose, median (IQR)	5.50 (5.00-6.40)	5.80 (5.00-7.10)	5.50 (5.00-6.40)	0.359
Creatinine, median (IQR)	86.00 (71.00-108.00)	83.00 (71.00-99.00)	86.00 (71.00-108.00)	0.033
Sodium, mean (SD)	138.30 (4.23)	138.38 (3.71)	138.30 (4.25)	0.761
Albumin, mean (SD)	36.63 (5.43)	36.65 (4.96)	36.63 (5.44)	0.952
Cholesterol, mean (SD)	4.85 (1.31)	5.01 (1.18)	4.85 (1.31)	0.077
INR, median (IQR)	1.04 (0.98-1.13)	1.01 (0.96-1.09)	1.04 (0.98-1.13)	**<0.001**
CRP, median (IQR)	11.00 (5.00-36.00)	5.00 (3.00-17.00)	12.00 (5.00-37.00)	**<0.001**
Hemoglobin, mean (SD)	-	-	-	-
White cell count, median (IQR)	8.80 (7.10-11.30)	8.20 (6.60-10.30)	8.80 (7.10-11.30)	**<0.001**
Platelet count, median (IQR)	245.00 (201.00-302.00)	242.50 (202.50-296.50)	245.00 (200.00-302.00)	0.536

Additional table 10 Patient characteristics stratified by whether or not data for **white cell count** levels were missing

	Total	Missing	Non-missing	p-value
Age, median (IQR)	80.00 (71.63-86.00)	81.00 (72.00-87.00)	80.00 (71.43-86.00)	0.490
Sex (% female)	5623 (52.01)	130 (48.51)	5493 (52.10)	0.246
Length of stay, median (IQR)	8.00 (3.00-17.00)	5.50 (2.00-13.00)	8.00 (3.10-17.00)	**<0.001**
mRS admission, median (IQR)	0.00 (0.00-2.00)	0.00 (0.00-1.00)	0.00 (0.00-2.00)	0.345
mRS discharge, median (IQR)	3.00 (1.00-6.00)	3.00 (1.00-6.00)	3.00 (1.00-6.00)	0.311
ΔmRS, median (IQR)	2.00 (0.00-3.00)	1.00 (0.00-3.00)	2.00 (0.00-3.00)	0.183
Charlson comorbidity index, median (IQR)	3.00 (1.00-4.00)	2.00 (1.00-3.00)	3.00 (1.00-4.00)	**<0.001**
Total NIHSS, median (IQR)	4.00 (1.00-10.00)	13.50 (1.00-25.50)	4.00 (1.00-10.00)	0.555
In-hospital mortality, n (%)	2042 (18.89)	65 (24.25)	1977 (18.75)	0.023
Antiplatelets at admission, n (%)	3610 (33.39)	83 (30.97)	3527 (33.45)	0.395
Antiplatelets at discharge, n (%)	7045 (65.16)	115 (42.91)	6930 (65.72)	**<0.001**
Anticoagulants at admission, n (%)	169 (1.56)	3 (1.12)	166 (1.57)	0.553
Anticoagulants at discharge, n (%)	434 (4.01)	12 (4.48)	422 (4.00)	0.695
Pneumonia, n (%)	2000 (18.50)	27 (10.07)	1973 (18.71)	**<0.001**
Asthma, n (%)	1011 (9.35)	22 (8.21)	989 (9.38)	0.516
Chronic obstructive pulmonary disease, n (%)	897 (8.30)	12 (4.48)	885 (8.39)	0.022
Transient ischemic attack, n (%)	559 (5.17)	13 (4.85)	546 (5.18)	0.811
Myocardial Infarction, n (%)	811 (7.50)	11 (4.10)	800 (7.59)	0.033
Hyperlipidemia, n (%)	1469 (13.59)	32 (11.94)	1437 (13.63)	0.426
Congenital heart disease, n (%)	3004 (27.78)	42 (15.67)	2962 (28.09)	**<0.001**
Peripheral vascular disease, n (%)	452 (4.18)	7 (2.61)	445 (4.22)	0.194
Heart failure, n (%)	1543 (14.27)	19 (7.09)	1524 (14.45)	**<0.001**
Atrial fibrillation, n (%)	3537 (32.71)	59 (22.01)	3478 (32.99)	**<0.001**
Hypertension, n (%)	6592 (60.97)	118 (44.03)	6474 (61.40)	**<0.001**

Cancers, n (%)	1771 (16.38)	43 (16.04)	1728 (16.39)	0.881
Chronic kidney disease, n (%)	719 (6.65)	10 (3.73)	709 (6.72)	0.052
Liver disease, n (%)	170 (1.57)	2 (0.75)	168 (1.59)	0.271
Random plasma glucose, median (IQR)	6.30 (5.50-7.80)	6.00 (5.10-7.40)	6.30 (5.50-7.80)	0.434
Fasting glucose, median (IQR)	5.50 (5.00-6.40)	5.80 (4.80-7.10)	5.50 (5.00-6.40)	0.817
Creatinine, median (IQR)	86.00 (71.00-108.00)	82.00 (71.00-99.00)	86.00 (71.00-108.00)	0.401
Sodium, mean (SD)	138.30 (4.23)	138.00 (2.69)	138.30 (4.24)	0.726
Albumin, mean (SD)	36.63 (5.43)	39.09 (4.58)	36.63 (5.43)	0.033
Cholesterol, mean (SD)	4.85 (1.31)	4.96 (1.18)	4.85 (1.31)	0.723
INR, median (IQR)	1.04 (0.98-1.13)	1.02 (0.97-1.04)	1.04 (0.98-1.13)	0.299
CRP, median (IQR)	11.00 (5.00-36.00)	5.00 (2.00-6.00)	11.00 (5.00-36.00)	0.004
Hemoglobin, mean (SD)	134.61 (19.57)	-	134.61 (19.57)	-
White cell count, median (IQR)	-	-	-	-
Platelet count, median (IQR)	245.00 (201.00-302.00)	-	245.00 (201.00-302.00)	-

Additional table 11 Patient characteristics stratified by whether or not data for **platelet** levels were missing

	Total	Missing	Non-missing	p-value
Age, median (IQR)	80.00 (71.63-86.00)	81.00 (72.00-87.00)	80.00 (71.50-86.00)	0.670
Sex (% female)	5623 (52.01)	138 (48.76)	5485 (52.09)	0.268
Length of stay, median (IQR)	8.00 (3.00-17.00)	5.00 (2.00-13.00)	8.00 (3.12-17.00)	**<0.001**
mRS admission, median (IQR)	0.00 (0.00-2.00)	0.00 (0.00-1.00)	0.00 (0.00-2.00)	0.346
mRS discharge, median (IQR)	3.00 (1.00-6.00)	3.00 (1.00-6.00)	3.00 (1.00-6.00)	0.413
ΔmRS, median (IQR)	2.00 (0.00-3.00)	1.00 (0.00-3.00)	2.00 (0.00-3.00)	0.171
Charlson comorbidity index, median (IQR)	3.00 (1.00-4.00)	2.00 (1.00-3.00)	3.00 (1.00-4.00)	**<0.001**
Total NIHSS, median (IQR)	4.00 (1.00-10.00)	13.50 (1.00-25.50)	4.00 (1.00-10.00)	0.555
In-hospital mortality, n (%)	2042 (18.89)	71 (25.09)	1971 (18.72)	0.007
Antiplatelets at admission, n (%)	3610 (33.39)	86 (30.39)	3524 (33.47)	0.278
Antiplatelets at discharge, n (%)	7045 (65.16)	121 (42.76)	6924 (65.76)	**<0.001**
Anticoagulants at admission, n (%)	169 (1.56)	3 (1.06)	166 (1.58)	0.489
Anticoagulants at discharge, n (%)	434 (4.01)	12 (4.24)	422 (4.01)	0.844
Pneumonia, n (%)	2000 (18.50)	28 (9.89)	1972 (18.73)	**<0.001**
Asthma, n (%)	1011 (9.35)	22 (7.77)	989 (9.39)	0.356
Chronic obstructive pulmonary disease, n (%)	897 (8.30)	13 (4.59)	884 (8.40)	0.022
Transient ischemic attack, n (%)	559 (5.17)	13 (4.59)	546 (5.19)	0.657
Myocardial Infarction, n (%)	811 (7.50)	11 (3.89)	800 (7.60)	0.019
Hyperlipidemia, n (%)	1469 (13.59)	33 (11.66)	1436 (13.64)	0.338
Congenital heart disease, n (%)	3004 (27.78)	43 (15.19)	2961 (28.12)	**<0.001**
Peripheral vascular disease, n (%)	452 (4.18)	9 (3.18)	443 (4.21)	0.394
Heart failure, n (%)	1543 (14.27)	21 (7.42)	1522 (14.46)	**<0.001**
Atrial fibrillation, n (%)	3537 (32.71)	61 (21.55)	3476 (33.01)	**<0.001**
Hypertension, n (%)	6592 (60.97)	125 (44.17)	6467 (61.42)	**<0.001**

Cancers, n (%)	1771 (16.38)	44 (15.55)	1727 (16.40)	0.701
Chronic kidney disease, n (%)	719 (6.65)	12 (4.24)	707 (6.71)	0.099
Liver disease, n (%)	170 (1.57)	3 (1.06)	167 (1.59)	0.483
Random plasma glucose, median (IQR)	6.30 (5.50-7.80)	6.00 (5.30-7.40)	6.30 (5.50-7.80)	0.508
Fasting glucose, median (IQR)	5.50 (5.00-6.40)	5.30 (4.80-7.10)	5.50 (5.00-6.40)	0.800
Creatinine, median (IQR)	86.00 (71.00-108.00)	87.00 (69.00-100.00)	86.00 (71.00-108.00)	0.610
Sodium, mean (SD)	138.30 (4.23)	138.00 (3.17)	138.30 (4.24)	0.655
Albumin, mean (SD)	36.63 (5.43)	37.76 (6.56)	36.63 (5.42)	0.206
Cholesterol, mean (SD)	4.85 (1.31)	5.00 (1.03)	4.85 (1.31)	0.556
INR, median (IQR)	1.04 (0.98-1.13)	1.03 (0.98-1.09)	1.04 (0.98-1.13)	0.614
CRP, median (IQR)	11.00 (5.00-36.00)	5.50 (5.00-11.00)	11.00 (5.00-36.00)	0.083
Hemoglobin, mean (SD)	134.61 (19.57)	130.93 (23.83)	134.61 (19.57)	0.467
White cell count, median (IQR)	8.80 (7.10-11.30)	10.40 (7.00-11.70)	8.80 (7.10-11.30)	0.682
Platelet count, median (IQR)	-	-	-	-

Additional table 12 Patient characteristics stratified by whether or not data for **OCSP classification** were missing

	Total	Missing	Non-missing	p-value
Age, median (IQR)	80.00 (71.63-86.00)	79.00 (70.00-86.00)	80.00 (72.00-86.00)	0.100
Sex (% female)	5623 (52.01)	340 (48.30)	5283 (52.27)	0.041
Length of stay, median (IQR)	8.00 (3.00-17.00)	6.00 (3.00-14.00)	8.00 (3.19-17.00)	**<0.001**
mRS admission, median (IQR)	0.00 (0.00-2.00)	0.00 (0.00-1.00)	0.00 (0.00-2.00)	0.352
mRS discharge, median (IQR)	3.00 (1.00-6.00)	2.00 (1.00-4.00)	3.00 (1.00-6.00)	**<0.001**
ΔmRS, median (IQR)	2.00 (0.00-3.00)	1.00 (0.00-3.00)	2.00 (1.00-3.00)	**<0.001**
Charlson comorbidity index, median (IQR)	3.00 (1.00-4.00)	3.00 (1.00-4.00)	3.00 (1.00-4.00)	0.776
Total NIHSS, median (IQR)	4.00 (1.00-10.00)	4.00 (2.00-13.00)	4.00 (1.00-10.00)	0.095
In-hospital mortality, n (%)	2042 (18.89)	91 (12.93)	1951 (19.30)	**<0.001**
Antiplatelets at admission, n (%)	3610 (33.39)	219 (31.11)	3391 (33.55)	0.184
Antiplatelets at discharge, n (%)	7045 (65.16)	427 (60.65)	6618 (65.47)	0.009
Anticoagulants at admission, n (%)	169 (1.56)	11 (1.56)	158 (1.56)	0.999
Anticoagulants at discharge, n (%)	434 (4.01)	64 (9.09)	370 (3.66)	**<0.001**
Pneumonia, n (%)	2000 (18.50)	145 (20.60)	1855 (18.35)	0.138
Asthma, n (%)	1011 (9.35)	65 (9.23)	946 (9.36)	0.912
Chronic obstructive pulmonary disease, n (%)	897 (8.30)	68 (9.66)	829 (8.20)	0.175
Transient ischemic attack, n (%)	559 (5.17)	43 (6.11)	516 (5.10)	0.245
Myocardial Infarction, n (%)	811 (7.50)	69 (9.80)	742 (7.34)	0.017
Hyperlipidemia, n (%)	1469 (13.59)	124 (17.61)	1345 (13.31)	0.001
Congenital heart disease, n (%)	3004 (27.78)	188 (26.70)	2816 (27.86)	0.508
Peripheral vascular disease, n (%)	452 (4.18)	21 (2.98)	431 (4.26)	0.101
Heart failure, n (%)	1543 (14.27)	86 (12.22)	1457 (14.41)	0.107
Atrial fibrillation, n (%)	3537 (32.71)	244 (34.66)	3293 (32.58)	0.255
Hypertension, n (%)	6592 (60.97)	461 (65.48)	6131 (60.65)	0.011

Cancers, n (%)	1771 (16.38)	145 (20.60)	1626 (16.09)	0.002
Chronic kidney disease, n (%)	719 (6.65)	73 (10.37)	646 (6.39)	**<0.001**
Liver disease, n (%)	170 (1.57)	18 (2.56)	152 (1.50)	0.030
Random plasma glucose, median (IQR)	6.30 (5.50-7.80)	6.40 (5.50-7.80)	6.30 (5.50-7.80)	0.498
Fasting glucose, median (IQR)	5.50 (5.00-6.40)	5.60 (5.00-7.30)	5.50 (5.00-6.40)	0.514
Creatinine, median (IQR)	86.00 (71.00-108.00)	83.00 (70.00-103.50)	86.00 (71.00-108.00)	0.003
Sodium, mean (SD)	138.30 (4.23)	137.90 (3.75)	138.33 (4.27)	0.011
Albumin, mean (SD)	36.63 (5.43)	35.43 (5.31)	36.71 (5.43)	**<0.001**
Cholesterol, mean (SD)	4.85 (1.31)	4.88 (1.36)	4.85 (1.31)	0.586
INR, median (IQR)	1.04 (0.98-1.13)	1.04 (0.98-1.15)	1.04 (0.98-1.12)	0.020
CRP, median (IQR)	11.00 (5.00-36.00)	7.00 (3.00-23.00)	12.00 (5.00-37.00)	**<0.001**
Hemoglobin, mean (SD)	134.61 (19.57)	134.53 (19.40)	134.61 (19.59)	0.914
White cell count, median (IQR)	8.80 (7.10-11.30)	8.80 (7.20-11.00)	8.80 (7.10-11.30)	0.723
Platelet count, median (IQR)	245.00 (201.00-302.00)	242.00 (189.50-302.00)	245.00 (201.00-302.00)	0.113

Additional table 13 Patient characteristics stratified by whether or not data for **pre-stroke mRS** scores were missing

	Total	Missing	Non-missing	p-value
Age, median (IQR)	80.00 (71.63-86.00)	82.00 (76.00-88.00)	80.00 (71.00-86.00)	**<0.001**
Sex (% female)	5623 (52.01)	334 (55.02)	5289 (51.83)	0.126
Length of stay, median (IQR)	8.00 (3.00-17.00)	11.00 (4.00-24.00)	8.00 (3.00-17.00)	**<0.001**
mRS admission, median (IQR)	-	-	-	-
mRS discharge, median (IQR)	3.00 (1.00-6.00)	6.00 (0.00-6.00)	3.00 (1.00-5.00)	**<0.001**
ΔmRS, median (IQR)	2.00 (0.00-3.00)	-	2.00 (0.00-3.00)	-
Charlson comorbidity index, median (IQR)	3.00 (1.00-4.00)	3.00 (1.00-4.00)	3.00 (1.00-4.00)	0.489
Total NIHSS, median (IQR)	4.00 (1.00-10.00)	4.00 (0.00-7.00)	4.00 (1.00-10.00)	0.547
In-hospital mortality, n (%)	2042 (18.89)	255 (42.01)	1787 (17.51)	**<0.001**
Antiplatelets at admission, n (%)	3610 (33.39)	136 (22.41)	3474 (34.04)	**<0.001**
Antiplatelets at discharge, n (%)	7045 (65.16)	160 (26.36)	6885 (67.47)	**<0.001**
Anticoagulants at admission, n (%)	169 (1.56)	0 (0.00)	169 (1.66)	0.001
Anticoagulants at discharge, n (%)	434 (4.01)	2 (0.33)	432 (4.23)	**<0.001**
Pneumonia, n (%)	2000 (18.50)	155 (25.54)	1845 (18.08)	**<0.001**
Asthma, n (%)	1011 (9.35)	58 (9.56)	953 (9.34)	0.859
Chronic obstructive pulmonary disease, n (%)	897 (8.30)	60 (9.88)	837 (8.20)	0.144
Transient ischemic attack, n (%)	559 (5.17)	28 (4.61)	531 (5.20)	0.523
Myocardial Infarction, n (%)	811 (7.50)	47 (7.74)	764 (7.49)	0.816
Hyperlipidemia, n (%)	1469 (13.59)	47 (7.74)	1422 (13.93)	**<0.001**
Congenital heart disease, n (%)	3004 (27.78)	162 (26.69)	2842 (27.85)	0.535
Peripheral vascular disease, n (%)	452 (4.18)	31 (5.11)	421 (4.13)	0.240
Heart failure, n (%)	1543 (14.27)	109 (17.96)	1434 (14.05)	0.008
Atrial fibrillation, n (%)	3537 (32.71)	192 (31.63)	3345 (32.78)	0.558
Hypertension, n (%)	6592 (60.97)	299 (49.26)	6293 (61.67)	**<0.001**

Cancers, n (%)	1771 (16.38)	112 (18.45)	1659 (16.26)	0.156
Chronic kidney disease, n (%)	719 (6.65)	43 (7.08)	676 (6.62)	0.659
Liver disease, n (%)	170 (1.57)	10 (1.65)	160 (1.57)	0.878
Random plasma glucose, median (IQR)	6.30 (5.50-7.80)	6.70 (5.60-8.40)	6.30 (5.50-7.80)	0.015
Fasting glucose, median (IQR)	5.50 (5.00-6.40)	5.70 (5.00-6.50)	5.50 (5.00-6.40)	0.490
Creatinine, median (IQR)	86.00 (71.00-108.00)	92.00 (73.00-120.00)	86.00 (71.00-107.00)	**<0.001**
Sodium, mean (SD)	138.30 (4.23)	138.25 (4.76)	138.31 (4.20)	0.776
Albumin, mean (SD)	36.63 (5.43)	34.57 (6.83)	36.74 (5.32)	**<0.001**
Cholesterol, mean (SD)	4.85 (1.31)	4.64 (1.18)	4.86 (1.31)	0.010
INR, median (IQR)	1.04 (0.98-1.13)	1.07 (1.00-1.21)	1.04 (0.98-1.12)	**<0.001**
CRP, median (IQR)	11.00 (5.00-36.00)	29.00 (11.00-75.00)	11.00 (5.00-34.00)	**<0.001**
Hemoglobin, mean (SD)	134.61 (19.57)	128.08 (20.81)	134.97 (19.44)	**<0.001**
White cell count, median (IQR)	8.80 (7.10-11.30)	10.10 (7.60-13.30)	8.80 (7.10-11.20)	**<0.001**
Platelet count, median (IQR)	245.00 (201.00-302.00)	253.00 (200.50-321.00)	245.00 (201.00-301.00)	0.067

Additional table 14 Patient characteristics stratified by whether or not data for **post-stroke mRS** scores were missing

	Total	Missing	Non-missing	p-value
Age, median (IQR)	80.00 (71.63-86.00)	79.00 (71.00-85.00)	81.00 (72.00-87.00)	**<0.001**
Sex (% female)	5623 (52.01)	1648 (52.20)	3975 (51.93)	0.795
Length of stay, median (IQR)	8.00 (3.00-17.00)	12.00 (6.00-23.00)	6.00 (3.00-15.00)	**<0.001**
mRS admission, median (IQR)	0.00 (0.00-2.00)	0.00 (0.00-1.00)	0.00 (0.00-2.00)	**<0.001**
mRS discharge, median (IQR)	-	-	-	-
ΔmRS, median (IQR)	2.00 (0.00-3.00)	-	2.00 (0.00-3.00)	-
Charlson comorbidity index, median (IQR)	3.00 (1.00-4.00)	3.00 (1.00-4.00)	2.00 (1.00-4.00)	**<0.001**
Total NIHSS, median (IQR)	4.00 (1.00-10.00)	4.00 (3.00-5.00)	4.00 (1.00-10.00)	0.908
In-hospital mortality, n (%)	2042 (18.89)	0 (0.00)	2042 (26.68)	**<0.001**
Antiplatelets at admission, n (%)	3610 (33.39)	1186 (37.57)	2424 (31.67)	**<0.001**
Antiplatelets at discharge, n (%)	7045 (65.16)	2399 (75.99)	4646 (60.69)	**<0.001**
Anticoagulants at admission, n (%)	169 (1.56)	0 (0.00)	169 (2.21)	**<0.001**
Anticoagulants at discharge, n (%)	434 (4.01)	1 (0.03)	433 (5.66)	**<0.001**
Pneumonia, n (%)	2000 (18.50)	252 (7.98)	1748 (22.83)	**<0.001**
Asthma, n (%)	1011 (9.35)	239 (7.57)	772 (10.08)	**<0.001**
Chronic obstructive pulmonary disease, n (%)	897 (8.30)	183 (5.80)	714 (9.33)	**<0.001**
Transient ischemic attack, n (%)	559 (5.17)	155 (4.91)	404 (5.28)	0.432
Myocardial Infarction, n (%)	811 (7.50)	170 (5.38)	641 (8.37)	**<0.001**
Hyperlipidemia, n (%)	1469 (13.59)	259 (8.20)	1210 (15.81)	**<0.001**
Congenital heart disease, n (%)	3004 (27.78)	768 (24.33)	2236 (29.21)	**<0.001**
Peripheral vascular disease, n (%)	452 (4.18)	128 (4.05)	324 (4.23)	0.674
Heart failure, n (%)	1543 (14.27)	390 (12.35)	1153 (15.06)	**<0.001**
Atrial fibrillation, n (%)	3537 (32.71)	864 (27.37)	2673 (34.92)	**<0.001**
Hypertension, n (%)	6592 (60.97)	1696 (53.72)	4896 (63.96)	**<0.001**

Cancers, n (%)	1771 (16.38)	415 (13.15)	1356 (17.71)	**<0.001**
Chronic kidney disease, n (%)	719 (6.65)	96 (3.04)	623 (8.14)	**<0.001**
Liver disease, n (%)	170 (1.57)	29 (0.92)	141 (1.84)	**<0.001**
Random plasma glucose, median (IQR)	6.30 (5.50-7.80)	6.10 (5.40-7.50)	6.40 (5.60-8.00)	**<0.001**
Fasting glucose, median (IQR)	5.50 (5.00-6.40)	5.60 (5.00-6.50)	5.50 (5.00-6.40)	0.931
Creatinine, median (IQR)	86.00 (71.00-108.00)	89.00 (75.00-110.00)	84.00 (70.00-107.00)	**<0.001**
Sodium, mean (SD)	138.30 (4.23)	139.04 (4.04)	138.00 (4.27)	**<0.001**
Albumin, mean (SD)	36.63 (5.43)	38.54 (5.35)	35.85 (5.26)	**<0.001**
Cholesterol, mean (SD)	4.85 (1.31)	4.92 (1.32)	4.82 (1.30)	0.003
INR, median (IQR)	1.04 (0.98-1.13)	1.03 (0.97-1.12)	1.04 (0.98-1.13)	**<0.001**
CRP, median (IQR)	11.00 (5.00-36.00)	17.00 (8.00-42.00)	9.00 (4.00-33.00)	**<0.001**
Hemoglobin, mean (SD)	134.61 (19.57)	135.72 (18.77)	134.13 (19.89)	**<0.001**
White cell count, median (IQR)	8.80 (7.10-11.30)	9.00 (7.30-11.30)	8.80 (7.10-11.30)	0.006
Platelet count, median (IQR)	245.00 (201.00-302.00)	251.00 (207.00-306.00)	243.00 (197.50-300.00)	<0.001

Additional table 15 Patient characteristics stratified by whether or not data for **NIHSS** scores were missing

	Total	Missing	Non-missing	p-value
Age, median (IQR)	80.00 (71.63-86.00)	80.00 (72.00-86.00)	79.99 (69.85-86.89)	0.371
Sex (% female)	5623 (52.01)	4882 (52.55)	741 (48.69)	0.005
Length of stay, median (IQR)	8.00 (3.00-17.00)	8.00 (4.00-18.00)	5.20 (2.23-13.77)	**<0.001**
mRS admission, median (IQR)	0.00 (0.00-2.00)	0.00 (0.00-2.00)	0.00 (0.00-1.00)	0.233
mRS discharge, median (IQR)	3.00 (1.00-6.00)	3.00 (1.00-6.00)	3.00 (1.00-4.00)	**<0.001**
ΔmRS, median (IQR)	2.00 (0.00-3.00)	2.00 (0.00-4.00)	2.00 (1.00-3.00)	0.111
Charlson comorbidity index, median (IQR)	3.00 (1.00-4.00)	3.00 (1.00-4.00)	2.00 (1.00-4.00)	0.204
Total NIHSS, median (IQR)	-	-	-	-
In-hospital mortality, n (%)	2042 (18.89)	1855 (19.97)	187 (12.29)	**<0.001**
Antiplatelets at admission, n (%)	3610 (33.39)	3609 (38.85)	1 (0.07)	**<0.001**
Antiplatelets at discharge, n (%)	7045 (65.16)	6087 (65.52)	958 (62.94)	0.050
Anticoagulants at admission, n (%)	169 (1.56)	21 (0.23)	148 (9.72)	**<0.001**
Anticoagulants at discharge, n (%)	434 (4.01)	154 (1.66)	280 (18.40)	**<0.001**
Pneumonia, n (%)	2000 (18.50)	1670 (17.98)	330 (21.68)	**<0.001**
Asthma, n (%)	1011 (9.35)	838 (9.02)	173 (11.37)	0.004
Chronic obstructive pulmonary disease, n (%)	897 (8.30)	724 (7.79)	173 (11.37)	**<0.001**
Transient ischemic attack, n (%)	559 (5.17)	475 (5.11)	84 (5.52)	0.507
Myocardial Infarction, n (%)	811 (7.50)	686 (7.38)	125 (8.21)	0.255
Hyperlipidemia, n (%)	1469 (13.59)	1175 (12.65)	294 (19.32)	**<0.001**
Congenital heart disease, n (%)	3004 (27.78)	2586 (27.84)	418 (27.46)	0.764
Peripheral vascular disease, n (%)	452 (4.18)	394 (4.24)	58 (3.81)	0.437
Heart failure, n (%)	1543 (14.27)	1326 (14.27)	217 (14.26)	0.987
Atrial fibrillation, n (%)	3537 (32.71)	3031 (32.63)	506 (33.25)	0.633
Hypertension, n (%)	6592 (60.97)	5577 (60.03)	1015 (66.69)	**<0.001**

Cancers, n (%)	1771 (16.38)	1473 (15.86)	298 (19.58)	**<0.001**
Chronic kidney disease, n (%)	719 (6.65)	548 (5.90)	171 (11.24)	**<0.001**
Liver disease, n (%)	170 (1.57)	133 (1.43)	37 (2.43)	0.004
Random plasma glucose, median (IQR)	6.30 (5.50-7.80)	6.30 (5.50-7.80)	6.40 (5.60-8.00)	0.023
Fasting glucose, median (IQR)	5.50 (5.00-6.40)	5.50 (5.00-6.40)	5.65 (5.00-6.45)	0.847
Creatinine, median (IQR)	86.00 (71.00-108.00)	87.00 (71.00-109.00)	82.00 (69.00-103.00)	**<0.001**
Sodium, mean (SD)	138.30 (4.23)	138.41 (4.27)	137.67 (3.95)	**<0.001**
Albumin, mean (SD)	36.63 (5.43)	36.80 (5.52)	35.64 (4.71)	**<0.001**
Cholesterol, mean (SD)	4.85 (1.31)	4.87 (1.31)	4.78 (1.28)	0.023
INR, median (IQR)	1.04 (0.98-1.13)	1.04 (0.97-1.12)	1.06 (1.00-1.14)	**<0.001**
CRP, median (IQR)	11.00 (5.00-36.00)	13.00 (5.00-40.00)	6.00 (3.00-19.00)	**<0.001**
Hemoglobin, mean (SD)	134.61 (19.57)	134.57 (19.51)	134.80 (19.97)	0.673
White cell count, median (IQR)	8.80 (7.10-11.30)	8.80 (7.10-11.30)	8.80 (7.10-11.10)	0.509
Platelet count, median (IQR)	245.00 (201.00-302.00)	246.00 (201.00-303.00)	241.00 (195.00-294.00)	0.008
