# Supplementary material for: The individual and combined impacts of pre-existing diabetes and dementia on ischemic stroke outcomes: a registry-based cohort study
Source: BMC Cardiovasc Disord. 2024 Jul 30;24:396. doi: 10.1186/s12872-024-04050-3 (PMC11290225; doi:10.1186/s12872-024-04050-3)
Supplement: Supplementary file 4 — Additional file 4. [file 12872_2024_4050_MOESM4_ESM.pdf]

Additional file 4 Results of in-hospital outcomes analyses

	**No NIHSS adjustment**		**NIHSS adjustment**	
	OR [95% CI]	P value	OR [95% CI]	P value
In-hospital death				
No DM or dementia	1 (reference)		1 (reference)	
DM only	0.84 [0.71-1.00]	0.053	0.84 [0.71-1.00]	0.053
Dementia only	1.23 [0.90-1.69]	0.189	1.23 [0.90-1.68]	0.196
Both DM and dementia	1.14 [0.59-2.18]	0.695	1.13 [0.59-2.17]	0.711
LoS greater than median				
No DM or dementia	1 (reference)		1 (reference)	
DM only	1.12 [1.00-1.26]	0.052	1.12 [1.00-1.26]	0.052
Dementia only	1.31 [1.02-1.68]	0.031	1.31 [1.02-1.68]	0.031
Both DM and dementia	2.25 [1.34-3.76]	0.002	2.25 [1.34-3.77]	0.002
Excess disability				
Second *Δ*mRS tertile vs first *Δ*mRS tertile				
No DM or dementia	1 (reference)		1 (reference)	
DM only	0.99 [0.85-1.16]	0.905	0.99 [0.85-1.16]	0.907
Dementia only	1.26 [0.91-1.75]	0.168	1.26 [0.91-1.75]	0.169
Both DM and dementia	1.24 [0.65-2.39]	0.509	1.24 [0.65-2.39]	0.509
Third *Δ*mRS tertile vs first *Δ*mRS tertile				
No DM or dementia	1 (reference)		1 (reference)	
DM only	1.00 [0.84-1.19]	0.983	1.00 [0.84-1.19]	0.971
Dementia only	1.53 [1.05-2.24]	0.027	1.54 [1.05-2.25]	0.027
Both DM and dementia	1.61 [0.76-3.41]	0.218	1.61 [0.76-3.42]	0.213

Models were adjusted for age, sex, OCSP classification, mRS, comorbidities (pneumonia, asthma, COPD, transient ischemic attack [TIA], myocardial infarction, hyperlipidemia, peripheral vascular disease, heart failure, atrial fibrillation, hypertension, cancers, chronic kidney disease, liver disease, and hemorrhagic stroke, and other types of stroke), antithrombotic medications, and relevant biochemical and hematological measurements on admission (random plasma glucose, creatinine, sodium, hemoglobin, white cell count, and platelets);

DM indicates diabetes mellitus, NIHSS, National Institutes of Health Stroke Scale; mRS, modified Rankin scale; LoS, length of stay; OR, odds ratio; CI, confidence interval
