# Supplementary material for: The individual and combined impacts of pre-existing diabetes and dementia on ischemic stroke outcomes: a registry-based cohort study
Source: BMC Cardiovasc Disord. 2024 Jul 30;24:396. doi: 10.1186/s12872-024-04050-3 (PMC11290225; doi:10.1186/s12872-024-04050-3)
Supplement: Supplementary file 5 — Additional file 5. [file 12872_2024_4050_MOESM5_ESM.pdf]

Additional file 5 Results of long-term outcomes analyses

Mortality						
	Post-discharge deaths (%)	N	**NIHSS adjustment**		**No NIHSS adjustment**	
			HR [95% CI]	P value	HR [95% CI]	P value
No dementia or DM	2365 (34.2)	6907	1 (reference)		1 (reference)	
DM only	571 (37.2)	1533	1.19 [1.08-1.32]	0.001	1.20 [1.08-1.32]	<0.001
Dementia only	97 (47.8)	203	1.71 [1.46-2.01]	<0.001	1.71 [1.47-2.00]	<0.001
Both DM and dementia	24 (49.0)	49	1.76 [1.33-2.37]	<0.001	1.76 [1.32-2.34]	<0.001
Recurrence						
	Recurrent stroke	N	HR [95% CI]	P value	HR [95% CI]	P value
No dementia or DM	617 (9.7)	6372	1 (reference)		1 (reference)	
DM only	158 (11.5)	1379	1.25 [1.06-1.49]	0.010	1.25 [1.05-1.49]	0.010
Dementia only	13 (6.9)	188	1.59 [1.15-2.20]	0.005	1.59 [1.15-2.20]	0.005
Both DM and dementia	1 (2.6)	39	2.06 [1.12-3.77]	0.019	2.06 [1.13-3.77]	0.019

Results are yielded from Cox regression models, which were adjusted for age, sex, OCSP classification, mRS, comorbidities (pneumonia, asthma, COPD, transient ischemic attack [TIA], myocardial infarction, hyperlipidemia, peripheral vascular disease, heart failure, atrial fibrillation, hypertension, cancers, chronic kidney disease, liver disease, and hemorrhagic stroke, and other types of stroke), antithrombotic medications, and relevant biochemical and hematological measurements at discharge (random plasma glucose, creatinine, sodium, hemoglobin, white cell count, and platelets);
DM indicates diabetes mellitus, HR, hazard ratio; CI, confidence interval;
Median follow-up was 5.5 and 3.8 years for mortality and recurrence, respectively.
