# Supplementary material for: The individual and combined impacts of pre-existing diabetes and dementia on ischemic stroke outcomes: a registry-based cohort study
Source: BMC Cardiovasc Disord. 2024 Jul 30;24:396. doi: 10.1186/s12872-024-04050-3 (PMC11290225; doi:10.1186/s12872-024-04050-3)
Supplement: Supplementary file 6 — Additional file 6. [file 12872_2024_4050_MOESM6_ESM.pdf]

Additional file 6 Results of in-hospital outcomes analyses with interaction terms

	OR [95% CI]	P value
In-hospital death		
No DM or dementia	1 (reference)	
DM only	0.84 [0.71-1.00]	0.053
Dementia only	1.23 [0.90-1.69]	0.189
DM and dementia	1.10 [0.53-2.28]	0.792
LoS greater than median		
No DM or dementia	1 (reference)	
DM only	1.12 [1.00-1.26]	0.052
Dementia only	1.31 [1.02-1.68]	0.031
DM and dementia	1.52 [0.86-2.71]	0.149
Excess disability		
Second ΔmRS tertile vs first ΔmRS tertile		
No DM or dementia	1 (reference)	
DM only	0.99 [0.85-1.16]	0.905
Dementia only	1.26 [0.91-1.75]	0.168
DM and dementia	1.03 [0.52-2.05]	0.928
Third ΔmRS tertile vs first ΔmRS tertile		
No DM or dementia	1 (reference)	
DM only	1.00 [0.84-1.19]	0.983
Dementia only	1.53 [1.05-2.24]	0.027
DM and dementia	1.09 [0.47-2.53]	0.848

This table reports the odds ratios yielded from the logistic regression models that include diabetes and dementia as interaction terms and with no NIHSS adjustments; models were adjusted for age, sex, OCSP classification, mRS, comorbidities (pneumonia, asthma, COPD, transient ischemic attack [TIA], myocardial infarction, hyperlipidemia, peripheral vascular disease, heart failure, atrial fibrillation, hypertension, cancers, chronic kidney disease, liver disease, and hemorrhagic stroke, and other types of stroke), antithrombotic medications, and relevant biochemical and hematological measurements on admission (random plasma glucose, creatinine, sodium, hemoglobin, white cell count, and platelets);

DM indicates diabetes mellitus, NIHSS, National Institutes of Health Stroke Scale; mRS, modified Rankin scale; LoS, length of stay; OR, odds ratio; CI, confidence interval
