# Supplementary material for: The individual and combined impacts of pre-existing diabetes and dementia on ischemic stroke outcomes: a registry-based cohort study
Source: BMC Cardiovasc Disord. 2024 Jul 30;24:396. doi: 10.1186/s12872-024-04050-3 (PMC11290225; doi:10.1186/s12872-024-04050-3)
Supplement: Supplementary file 7 — Additional file 7. [file 12872_2024_4050_MOESM7_ESM.pdf]

**Additional file 7** Results of long-term outcomes analyses with interaction terms

| | HR [95% CI] | P value |
|---|---|---|
| Mortality | | |
| No DM or dementia | 1 (reference) | |
| DM only | 1.20 [1.08-1.32] | <0.001 |
| Dementia only | 1.71 [1.47-2.00] | <0.001 |
| DM and dementia | 0.85 [0.61-1.18] | 0.331 |

This table reports the hazard ratios yielded from the Cox regression model that include diabetes and dementia as interaction terms and with no NIHSS adjustments; the model was adjusted for age, sex, OCSP classification, mRS, recurrence, comorbidities (pneumonia, asthma, COPD, transient ischemic attack [TIA], myocardial infarction, hyperlipidemia, peripheral vascular disease, heart failure, atrial fibrillation, hypertension, cancers, chronic kidney disease, liver disease, and hemorrhagic stroke, and other types of stroke), antithrombotic medications, and relevant biochemical and hematological measurements at discharge (random plasma glucose, creatinine, sodium, hemoglobin, white cell count, and platelets);

DM indicates diabetes mellitus, HR, hazard ratio; CI, confidence interval
